# Supplementary material for: Genome-wide association analysis of anthracnose resistance in sorghum [Sorghum bicolor (L.) Moench]
Source: PLoS One. 2021 Dec 20;16(12):e0261461. doi: 10.1371/journal.pone.0261461 (PMC8687563; doi:10.1371/journal.pone.0261461)
Supplement: Attachment — Submitted filename: PONE-D-21-10117_R1.pdf [file pone.0261461.s004.pdf]

PLOS ONE

Genome-wide association analysis of anthracnose resistance in sorghum [Sorghum bicolor (L.) Moench]

--Manuscript Draft--

**Manuscript Number:**	PONE-D-21-10117R1
**Article Type:**	Research Article
**Full Title:**	Genome-wide association analysis of anthracnose resistance in sorghum [Sorghum bicolor (L.) Moench]
**Short Title:**	Genome-wide association analysis in sorghum anthracnose resistance
**Corresponding Author:**	Girma Mengistu Digafe, Ph.D.<br>University of KwaZulu-Natal - Pietermaritzburg Campus<br>pietermaritzburg, SOUTH AFRICA
**Keywords:**	anthracnose resistance, DArTseq technology, marker-trait association, QTL
**Abstract:**	In warm-humid ago-ecologies of the world, sorghum [Sorghum bicolor (L.) Moench] production is severely affected by anthracnose disease caused by Colletotrichum sublineolum Henn. New sources of anthracnose resistance should be identified to introgress novel genes into susceptible varieties in resistance breeding programs. The objective of this study was to determine genome wide association of Diversity Arrays Technology Sequencing (DArTseq) based single nucleotide polymorphisms (SNP) markers and anthracnose resistance genes in diverse sorghum population for resistance breeding. Three hundred sixty-six sorghum populations were assessed for anthracnose resistance in three seasons in western Ethiopia using artificial inoculation. Data on anthracnose severity and the relative area under disease progress curve were computed. Furthermore, the test populations were genotyped using SNP markers with DArTseq protocol. Population structure analysis and genome-wide association mapping were undertaken based on 11,643 SNPs with <10% missing data. The evaluated population was grouped into eight distinct genetic clusters. A total of eight significant (P < 0.001) marker-trait associations (MTAs) were detected explaining 4.86-15.9% of the phenotypic variation for anthracnose resistance out of which four were above the cutoff point. The significant MTAs present in the assessed sorghum population are useful for marker-assisted selection (MAS) in anthracnose resistance breeding programs and for gene and quantitative trait loci (QTL) mapping.
**Order of Authors:**	Girma Mengistu Digafe, Ph.D.
	Hussein Shimelis
	Ermias Assefa
	Dagnachew Lule
**Response to Reviewers:**	Manuscript number, PONE-D-21-10117<br><br>Reviewers' comments:<br><br>Reviewer's Responses to Questions<br>Comments to the Author<br><br>1. Is the manuscript technically sound, and do the data support the conclusions?<br><br>The manuscript must describe a technically sound piece of scientific research with data that supports the conclusions. Experiments must have been conducted rigorously, with appropriate controls, replication, and sample sizes. The conclusions must be drawn appropriately based on the data presented.<br>Reviewer #1: Partly<br>Reviewer #2: Yes<br>Response 1. Thank you for the constructive feedback. The manuscript is based on integrated data sets through field-based phenomic and genomic analyses which supported the final conclusions and recommendations. The field experiments were conducted through rigorous data location across three consecutive seasons involving

Powered by Editorial Manager® and ProduXion Manager® from Aries Systems Corporation

sufficiently large number of sorghum germplasm collections and anthracnose susceptible and resistant standard controls. The conclusions drawn from the study were in synchronization with the phenomic and genomic data and sound genetic analyses.

____

2. Has the statistical analysis been performed appropriately and rigorously?
Reviewer #1: Yes
Reviewer #2: Yes
Response 2. Thank you for the comment. The statistical analysis has been performed appropriately and rigorously.

____

3. Have the authors made all data underlying the findings in their manuscript fully available?

The PLOS Data policy requires authors to make all data underlying the findings described in their manuscript fully available without restriction, with rare exception (please refer to the Data Availability Statement in the manuscript PDF file). The data should be provided as part of the manuscript or its supporting information, or deposited to a public repository. For example, in addition to summary statistics, the data points behind means, medians and variance measures should be available. If there are restrictions on publicly sharing data—e.g. participant privacy or use of data from a third party—those must be specified.

Reviewer #1: No
Reviewer #2: Yes
Response 3. The comment is accepted. Both data sets (genotypic and anthracnose severity data) are provided as a Supplementary Table 1 and 2.

____

4. Is the manuscript presented in an intelligible fashion and written in standard English?

PLOS ONE does not copyedit accepted manuscripts, so the language in submitted articles must be clear, correct, and unambiguous. Any typographical or grammatical errors should be corrected at revision, so please note any specific errors here.
Reviewer #1: No
Reviewer #2: Yes
Response 4. Thank you for the comments. The authors have checked the manuscript and typographical or grammatical errors are promptly fixed.____

5. Review Comments to the Author

Please use the space provided to explain your answers to the questions above. You may also include additional comments for the author, including concerns about dual publication, research ethics, or publication ethics. (Please upload your review as an attachment if it exceeds 20,000 characters)

Reviewer #1: Genome wide association analysis of anthracnose resistance in sorghum [Sorghum bicolor (L.) Moench]

This manuscript includes the anthracnose resistance evaluation of 366 Ethiopian accessions over three years and the genotyping characterization of these accessions based on DArT sequencing markers. Later the authors analyzed the population structure of this Ethiopian accessions and performed GWAS to identify anthracnose resistance loci.

Definitely, this manuscript has valuable information for sorghum breeding program in Ethiopia and other countries. However, the authors need to improve the manuscript to be accepted for publications. At this stage, the manuscript has many concerns that need to be addressed, and I suggest to the authors to resubmit the manuscript after its improvement.

Powered by Editorial Manager® and ProduXion Manager® from Aries Systems Corporation

	1- The English of the manuscript need to be improved. It has multiple typo errors and sentences that need to be rewrite for clarification. The use of number in reference needs to be verified in the sentences because many are difficult to read. Eg. Also [9] examined 114 recombinant inbred lines……<br><br>Response 1. As advised, the use of number(s) in the reference have been revised. This will ease to reading the references in the manuscript.<br>2- The evaluation of anthracnose is not well explained in the manuscript. They just mention is acording to Chala et al. 2012. They need to provide some information of how they did the scoring.<br>Response 2. The comment is accepted. We have added one paragraph.<br><br>3- Candidate genes were identified based in the SNPs position. They authors should explain the window size (20Kb upstream/downstream of the SNP) used to identity candidate genes.<br><br>Response 3. Thank you for the insight. This was achieved using the physical genome assembly of sorghum reference genome sequence, version 3.1.1 (https://phytozome.jgi.doe.gov/pz/portal.html#!info?alias=Org Sbicolor) serving as identifying candidate genes between 20 kb on either side of significant SNPs.<br><br>4- They authors should provide a supplementary table with the anthracnose score of each accession. Also make available the DArT genotyping.<br><br>Response 4. Data on anthracnose score for each accession and DArT genotyping are provided as Supplementary Table 1 and 2.<br><br>5- The authors never mentioned how many accessions were finally classified as resistant to anthracnose. I am wondering if they did a means compare analysis, contrast, or any other statistical analysis to classify an accession resistant to anthracnose.<br><br>Response 5. Thank you for the comment. 63 accessions were identified as moderately resistant to anthracnose disease. This is indicated in the Results section.<br><br>6- Population structure analysis is confuse. They first mention the optimal number is 8, then in the second paragraph they mention 10.<br><br>Response 6. The error is corrected, and the number of sub-populations are 8.<br><br>7- The marker trait association is confuse. In Mat and Met they indicate they will use the FDR p values less than 0.1 according to Benjamini and Hochberg. Then in results they mentioned eight significant SNPs and highlight that 4 were above the cutoff point. Do you have two threshold? You mentioned just one in Mat and Met.<br><br>Response 7. Thank you. We have added a description in Fig 4. A dash line represents the threshold from the FDR, and a blue line represents the significant threshold −Log10 (P) value.<br><br>8- The discussion is poor. Previous studies based on Ethiopian germplasm identified an anthracnose resistant locus in chromosome 9. How distance is the DArT markers from this locus?<br><br>Response 8. The distance from previously identified Ethiopian germplasm an anthracnose resistant locus in chromosome 9 is 360Kb.<br><br>9- The discussion don't include the population structure analysis of other Ethiopian germplasm. Eg. Cuevas et al. 2017 BMC Genomics, Girma et al. 2019, Menamo et al. 2021 TAG,<br><br>Response 9. Thank you for this input. We have revised as follows: Previous studies of population structure identified 11 genetic groups using 1,425 Ethiopian sorghum accessions [12]. In addition, a total of 318 Sudanese sorghum core collections were evaluated with 183,144 SNP markers using the model-based clustering method that

portrayed five subpopulations [11]. Furthermore, 940 diverse sorghum landraces of Ethiopia were assessed using 54,080 SNP markers that identified 12 subpopulations [48].

10 – Most of the discussion is centralized in candidate genes, instead of how to use the information for breeding and the conservation of sorghum germplasm.

Response 10. The anthracnose resistance genes identified in the present study will be validated across multiple seasons and locations to serves as ideal molecular markers for anthracnose resistance breeding programs. The novel genes could be pyramided into anthracnose susceptible sorghum lines through marker-assisted selection. A combinations of QTLs could render durable resistance to sorghum anthracnose.

11- Reference num 38 have an incorrect format. Last name must be first.
Response 11. Accepted. We have corrected the reference number 38 in the manuscript.

Reviewer #2: The manuscript entitled “Genome-wide association analysis of anthracnose resistance in sorghum [Sorghum bicolor (L.) Moench]” was written well and identified four novel markers (rs1887698, rs2681689, rs1884746 and rs100028710) associated with anthracnose resistance. Also by using the reference genome, authors identified the putative functional candidate genes annotated with genes in other species such as Arabidopsis and tomato. Population structure analysis with the clustering of lines and GWAS study for marker-trait association is well documented. However, the manuscript will be completed only if the identified markers are validated with additional study. To accelerate the breeding process for anthracnose resistance, only validated markers are more useful for large scale screening. Hence, authors are requested to provide the additional information on the identified markers’ validation.
Response. Thank you for the encouraging comment. Our next plan is to validate the identified four novel markers and implement marker-assisted breeding to stack the genes in a desirable genetic background.

---

6. PLOS authors have the option to publish the peer review history of their article (what does this mean?). If published, this will include your full peer review and any attached files.

Response 6. Yes

If you choose “no”, your identity will remain anonymous but your review may still be made public.

Do you want your identity to be public for this peer review? For information about this choice, including consent withdrawal, please see our Privacy Policy.

Reviewer #1: No
Reviewer #2: No

[NOTE: If reviewer comments were submitted as an attachment file, they will be attached to this email and accessible via the submission site. Please log into your account, locate the manuscript record, and check for the action link "View Attachments". If this link does not appear, there are no attachment files.]

While revising your submission, please upload your figure files to the Preflight Analysis and Conversion Engine (PACE) digital diagnostic tool, https://pacev2.apexcovantage.com/. PACE helps ensure that figures meet PLOS requirements. To use PACE, you must first register as a user. Registration is free. Then, login and navigate to the UPLOAD tab, where you will find detailed instructions on how to use the tool. If you encounter any issues or have any questions when using PACE, please email PLOS at figures@plos.org. Please note that Supporting Information files do not need this step.

Additional Information:	
**Question**	**Response**
**Financial Disclosure**<br><br>Enter a financial disclosure statement that describes the sources of funding for the work included in this submission. Review the submission guidelines for detailed requirements. View published research articles from *PLOS ONE* for specific examples.<br><br>This statement is required for submission and **will appear in the published article** if the submission is accepted. Please make sure it is accurate.<br><br>**Unfunded studies**<br>Enter: *The author(s) received no specific funding for this work.*<br><br>**Funded studies**<br>Enter a statement with the following details:<br>• Initials of the authors who received each award<br>• Grant numbers awarded to each author<br>• The full name of each funder<br>• URL of each funder website<br>• Did the sponsors or funders play any role in the study design, data collection and analysis, decision to publish, or preparation of the manuscript?<br>• **NO** - Include this sentence at the end of your statement: *The funders had no role in study design, data collection and analysis, decision to publish, or preparation of the manuscript.*<br>• **YES** - Specify the role(s) played.<br><br>* typeset	National Research Foundation (NRF)/South Africa for financial support for the research activity and the first author is postdoctoral in University of KwaZulu-Natal (UKZN)
**Competing Interests**<br><br>Use the instructions below to enter a competing interest statement for this submission. On behalf of all authors, disclose any competing interests that could be perceived to bias this work—acknowledging all financial support and any other relevant financial or non-financial competing interests.	The authors declare that they have no competing interests.

Powered by Editorial Manager® and ProduXion Manager® from Aries Systems Corporation

This statement is required for submission and **will appear in the published article** if the submission is accepted. Please make sure it is accurate and that any funding sources listed in your Funding Information later in the submission form are also declared in your Financial Disclosure statement.<br><br>View published research articles from *PLOS ONE* for specific examples.<br><br>**NO authors have competing interests**<br><br>Enter: *The authors have declared that no competing interests exist.*<br><br>**Authors with competing interests**<br><br>Enter competing interest details beginning with this statement:<br><br>*I have read the journal's policy and the authors of this manuscript have the following competing interests: [insert competing interests here]*<br><br>* typeset	
**Ethics Statement**<br><br>Enter an ethics statement for this submission. This statement is required if the study involved:<br><br>• Human participants<br>• Human specimens or tissue<br>• Vertebrate animals or cephalopods<br>• Vertebrate embryos or tissues<br>• Field research<br><br>Write "N/A" if the submission does not require an ethics statement.<br><br>General guidance is provided below. Consult the submission guidelines for detailed instructions. **Make sure that all information entered here is included in the**	No permission required activities done

**Methods section of the manuscript.**<br><br>**Format for specific study types**<br><br>**Human Subject Research (involving human participants and/or tissue)**<br>• Give the name of the institutional review board or ethics committee that approved the study<br>• Include the approval number and/or a statement indicating approval of this research<br>• Indicate the form of consent obtained (written/oral) or the reason that consent was not obtained (e.g. the data were analyzed anonymously)<br><br>**Animal Research (involving vertebrate animals, embryos or tissues)**<br>• Provide the name of the Institutional Animal Care and Use Committee (IACUC) or other relevant ethics board that reviewed the study protocol, and indicate whether they approved this research or granted a formal waiver of ethical approval<br>• Include an approval number if one was obtained<br>• If the study involved *non-human primates*, add *additional details* about animal welfare and steps taken to ameliorate suffering<br>• If anesthesia, euthanasia, or any kind of animal sacrifice is part of the study, include briefly which substances and/or methods were applied<br><br>**Field Research**<br><br>Include the following details if this study involves the collection of plant, animal, or other materials from a natural setting:<br>• Field permit number<br>• Name of the institution or relevant body that granted permission	
**Data Availability**<br><br>Authors are required to make all data underlying the findings described fully available, without restriction, and from the time of publication. PLOS allows rare exceptions to address legal and ethical concerns. See the PLOS Data Policy and FAQ for detailed information.	Yes - all data are fully available without restriction

A Data Availability Statement describing where the data can be found is required at submission. Your answers to this question constitute the Data Availability Statement and **will be published in the article**, if accepted.<br><br>**Important:** Stating 'data available on request from the author' is not sufficient. If your data are only available upon request, select 'No' for the first question and explain your exceptional situation in the text box.<br><br>Do the authors confirm that all data underlying the findings described in their manuscript are fully available without restriction?	
**Describe where the data may be found in full sentences. If you are copying our sample text, replace any instances of XXX with the appropriate details.**<br><br>• If the data are **held or will be held in a public repository**, include URLs, accession numbers or DOIs. If this information will only be available after acceptance, indicate this by ticking the box below. For example: *All XXX files are available from the XXX database (accession number(s) XXX, XXX.*).<br>• If the data are all contained **within the manuscript and/or Supporting Information files**, enter the following: *All relevant data are within the manuscript and its Supporting Information files.*<br>• If neither of these applies but you are able to provide **details of access elsewhere**, with or without limitations, please do so. For example:<br><br>*Data cannot be shared publicly because of [XXX]. Data are available from the XXX Institutional Data Access / Ethics Committee (contact via XXX) for researchers who meet the criteria for access to confidential data.*<br><br>*The data underlying the results*	All relevant data are within the manuscript and its Supporting Information files.

presented in the study are available from (include the name of the third party and contact information or URL).<br>• This text is appropriate if the data are owned by a third party and authors do not have permission to share the data.<br><br>* typeset	
Additional data availability information:	Tick here if the URLs/accession numbers/DOIs will be available only after acceptance of the manuscript for publication so that we can ensure their inclusion before publication.

Manuscript

Click here to access/download;Manuscript;Manucsript PONE-D-21-10117.docx

Genome-wide association analysis of anthracnose resistance in sorghum [*Sorghum bicolor* (L.) Moench]

Girma Mengistu[1,2]*, Hussein Shimelis[1], Ermias Assefa[3] and Dagnachew Lule[2]

1 University of KwaZulu-Natal, School of Agricultural, Earth and Environmental Sciences, Private Bag X01, Scottsville 3209, Pietermaritzburg, South Africa, 2 Oromia Agricultural Research Institute, P.O. Box 81265, Addis Ababa, Ethiopia, 3 Ethiopian Biotechnology Institute, P.O. Box 5954, Addis Ababa, Ethiopia

* germame2004@gmail.com

Abstract

In warm-humid ago-ecologies of the world, sorghum [*Sorghum bicolor* (L.) Moench] production is severely affected by anthracnose disease caused by *Colletotrichum sublineolum* Henn. New sources of anthracnose resistance should be identified to introgress novel genes into susceptible varieties in resistance breeding programs. The objective of this study was to determine genome wide association of Diversity Arrays Technology Sequencing (DArTseq) based single nucleotide polymorphisms (SNP) markers and anthracnose resistance genes in diverse sorghum population for resistance breeding. Three hundred sixty-six sorghum populations were assessed for anthracnose resistance in three seasons in western Ethiopia using artificial inoculation. Data on anthracnose severity and the relative area under disease progress curve were computed. Furthermore, the test populations were genotyped using SNP markers with DArTseq protocol. Population structure analysis and genome-wide association mapping were undertaken based on 11,643 SNPs with <10% missing data. The evaluated population was grouped into eight distinct genetic clusters. A total of eight significant ($P < 0.001$) marker-trait associations (MTAs) were detected explaining 4.86-15.9% of the phenotypic variation for anthracnose resistance out of which four were above the cutoff point. The significant MTAs present in the assessed sorghum population are useful for marker-assisted selection (MAS) in anthracnose resistance breeding programs and for gene and quantitative trait loci (QTL) mapping.

Keywords: anthracnose resistance, DArTseq technology, marker-trait association, QTL

Introduction

Sorghum [*Sorghum bicolor* (L.) Moench, 2n=2x=20] is an important cereal crop cultivated globally for multiple uses [1]. It is a mainstay crop in arid and semi-arid agro-ecologies due to its relatively higher drought tolerance compared with other common cereal crops such as maize and wheat. Sorghum production and productivity is affected by various constraints notably by biotic stresses such as diseases, weeds (*Striga* species) and insect pests. Anthracnose, grain mold, leaf blight, rust and smut are the most important diseases of sorghum, while stem borer, shoot fly, termites and birds are the common pests attacking the crop [2-4].

Sorghum anthracnose caused by the fungal pathogen *Colletotrichum sublineolum* Henn. (previously known as *C. graminicola* [Ces.] G.W. Wilson) is amongst the most important diseases of the crop. Anthracnose causes yield and quality losses in sorghum production. For instance, grain yield loss of 50-70% have been reported in susceptible sorghum varieties in the drier and humid agro-ecologies of Ethiopia [5, 6]. Development and deployment of anthracnose resistant sorghum varieties is an economic and environmentally friendly approach that could bolster sustainable sorghum production and productivity. Understanding the genetic basis and dissection of genes conditioning anthracnose resistance using genomic tools enhances gene introgression and marker-assisted selection [4, 7-10].

Genome-wide association study (GWAS) has been widely used to resolve complex genes controlling economic traits in crop plants including anthracnose resistance for effective breeding and genetic analysis [4, 7, 8, 11]. Genome-wide genetic analysis enables to delineate genomic regions such as markers, genes or quantitative trait loci (QTL) associated with key component

traits for marker-assisted breeding, gene discovery or gene introgression [7, 9]. Genome wide association analysis is dependent on marker-trait association (MTA) using representative markers and genetically diverse test populations which may include landraces, advanced breeding lines and improved cultivars [12, 13]. Identification of marker to trait associations will enhance gene introgression and selection gains through marker-assisted breeding.

Single nucleotide polymorphisms (SNP) is a marker of choice for genetic diversity analysis, characterization of genetic resources, cultivar identification, heterotic grouping, construction of high-resolution genetic maps, linkage disequilibrium-based association mapping and genetic diagnostics [14]. SNPs have been used in GWAS of various traits in sorghum including genetic analysis for resistance to diseases such as anthracnose [4, 7-9], grain mold [2], downy mildew, and head smut [4] and tolerance to drought [15]. Diversity Arrays Technology Sequencing (DArTseq) based single nucleotide polymorphisms (SNPs) is a powerful genomic tool used in GWAS of diverse agriculturally important crop traits [16].

Ethiopia is the center of origin and diversity of both cultivated and wild sorghum [17-20]. The country maintains more than 9000 sorghum accessions through the Ethiopian Biodiversity Institute (EBI) [12]. Sorghum genetic resources from Ethiopia have been used globally in genetic analysis and cultivar development programs [2, 12]. Some novel genes conditioning resistance to grain mold [2] and anthracnose [8] were reported amongst Ethiopian sorghum. The sorghum genetic resources maintained by the EBI should be systematically explored and used in improvement programs.

The sorghum genome was previously sequenced as a foundational database to guide genetic analysis and breeding [21]. Furthermore, various studies reported sorghum genomic regions associated with genes governing the inheritance of different traits such as anthracnose resistance [4, 7, 9, 10, 22], male sterility [12], flowering and plant height [12], heat tolerance [15] and grain polyphenol content [13]. Quantitative trait loci (QTL) conditioning the contents of protein, fat, and starch were reported in sorghum [23]. There are limited genomic studies that examined marker-trait associations including for anthracnose resistance with diverse and representative genetic pool of sorghum in Ethiopia.

The Bako areas in western parts of Ethiopia are known to maize and sorghum production. The areas are a known hotspot sites for sorghum anthracnose owing to its warm temperatures and high relative humidity [5]. To initiate anthracnose resistance breeding program a large number of sorghum genetic resources initially collected by the Ethiopian Biodiversity Institute (EBI) from nine regions in Ethiopia were provided by the Melkassa Agricultural Research Center. . The genetic resources preserved by Bako Agricultural Research Center should be further profiled using high density SNP markers and anthracnose resistance to identify and introgress novel genes into susceptible varieties by resistance breeding programs to enhance productivity of the crop. In light of the above background, the objective of this study was to determine genome wide association of Diversity Arrays Technology Sequencing based single nucleotide polymorphisms markers and anthracnose resistance genes in diverse sorghum population of Ethiopia for resistance breeding.

Materials and methods

Plant materials

The study used a population of 366 sorghum collections sourced from different geographical locations in Ethiopia including the Afar, Amhara, Benshangul Gumuz, Dire Dawa, Gambella, Oromiya, SNNP, Somali, and Tigray regions acquired from Melkassa Agricultural Research Center in Ethiopia. Three improved varieties ‘Gemedi’, ‘Geremew’ and ‘Btx623’ were included as comparative controls. Btx623 is anthracnose susceptible variety obtained from Texas A and M University, USA. The descriptions of the test accessions are presented in Table 1.

Study site and experimental design

The genotypes were evaluated for anthracnose resistance at Bako (9º6’ N; 37º9’ E) Agricultural Research Center (BARC) in Ethiopia. The center receives annual rainfall of 1,600 mm, while the mean maximum and minimum temperatures are 29 ºC and 13 ºC, respectively. The mean monthly relative humidity varies from 46 to 57%. The main rainy season ranges from May to October, with the most rain received in July and August. Short rains are also received during March to June. The trends of weather data for BARC during the study periods (2016-2018) are summarized in Fig 1. The soil type of the study site is nitosol. The genotypes were established using a 61 x 6 row by column incomplete block design with three replications. Each plot consisted of a single row of 2.1 m long with inter-row and intra-row spacing of 75 cm and 15 cm, respectively.

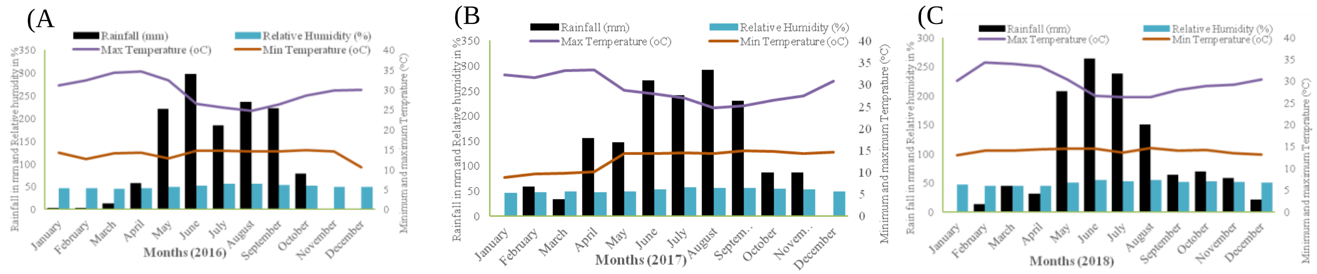

Figure 1. Rainfall, relative humidity, and minimum and maximum temperatures of Bako Agricultural Research Center in 2016 (A), 2017 (B) and 2018 (C). (Source: Bako Agricultural Research Center/Ethiopia)

Pathotyping for anthracnose reaction, data collection and analysis

Based on preliminary evaluations, single virulent anthracnose spore sourced from the Bako area was aseptically isolated, multiplied and used for inoculation of plants 45 days after sowing using the commonly used procedure [24]. The 366 genotypes were rigorously screened for anthracnose resistance at Bako for three seasons (2016–2018). Data on percentage severity of leaf area damaged by the anthracnose were recorded beginning from 15 days after inoculation five times at 10 days' intervals from five randomly tagged plants. The percentage of total leaf area of plants damaged by anthracnose were recorded following [25]. The final disease rating was measured 55 days after inoculation and was referred to as a final anthracnose severity (FAS). FAS was used to distinguish anthracnose reaction of lines based on the level of severity. Mean severity percentage values for each plot were used for data analysis.

Disease severity for anthracnose was used to calculate the area under disease progress curve (AUDPC). The AUDPC were calculated for each sorghum accession based on [26] as follows:

$$\mathrm{AUDPC} = \sum_{i=1}^{n-1} [(X_i + X_{i+1})/2]\,(t_{i+1} - t_i)$$

~~The final disease rating was measured 55 days after inoculation and was referred to as a final anthracnose severity (FAS)~~. The AUDPC values were converted into relative area under disease

progress curve (rAUDPC) as a ratio of the actual AUDPC of a sorghum entry to the AUDPC of a susceptible landrace (Acc#239182) across the three cropping seasons[illegible]he data were checked for normality, homogeneity of variance and validity for analysis of variance following the Bartlet test [27]. Data collected were analyzed using SAS computer software [28].

Table 1. List of 366 sorghum genotypes used in this study

**Collection regions or research centres**	**Accessions**
	Landraces
Afar	72564,72998, **73003**, 73006, 73007, **73008**, **73019**, **73026,** 73643, 73645, 206210, 206212
Amhara	69252, **70376**, 72443, 72467, **72474**, 72477, **72520**, **72524**, 72526, 72616, 73037, 73041, 73042, 73045, 73048, 73049, 73074, **73079**, **73095**, 75274, 75455, 200539, 210945, 210949, 210971, 210974, **211237**, **212640**, 213354, 214845, 214852, 226047, 226048, 226054, 226057, 228112, **228115**, 229887, 239154, 239179, 239180, 239182, **239184**, 239187, 239188, 239194, 239197, 239219, 239228, 239250, 242052, 243645, 243650, 243657
Benshangul	SC283-14, **ETSL100375**, **PML981475**, 229832
Dire Dawa	70742, 71161, 71180, 228840, 239114, **239115**, 239116, 239117, 239118, 239119, 239123, 239124, 239125, 239126, **239127**, 239129, **239131**, 239132, 239133, 239134, **239135**, 239137
Gambella	69372, 69412, 70027, 70028, 70051, 71569, 71570, 71571, 71574, 71623, 71624, 71625, 71628, 71631, 71635, 71642, 71643, 71644, 71648, 71653, 71654, 71656, 71658, 71698, **71700**, 71701, 71708, 71711, 71712, 71714, 71720, 74914, 200522, 201433, 206149, 206154, 211209, 211210, **222885**
Oromiya	9110, **9116**, 15830, **15832**, **15877**, 15890, 15897, 15904, **15908**, 15914, 15932, 15935, 15956, **16113**, 16116, 16133, **16135**, 16152, 16162, **16163**, 16168, 16171, 16173, 16176, 16177, 16180, 16206, 16208, 16212, 16213, 16440, 16450, 16451, 16477, 16487, 16489, 17518, 69534, **69540**, 69553, 70282, 70471, 70704, 70842, 70859, 70943, 70967, 70998, 71044, 71110, 71137, 71154, 71165, 71168, 71169, 71177, 71194, 71319, 71334, **71337**, 71363, 71372, 71374, 71392, **71395**, 71466, 71500, 71502, 71503, **71507**, 71513, 71516, 71524, **71544**, 71545, 71546, 71547, 71548, 71549, **71550**, 71551, 71553, 71555, 71556, 71557, 71558, 71559, 71560, 71562, **71563**, 75003, 75004, **75006**, 75114, 75115, **75118**, 75119, 75120, 75123, 75132, 75143, 75146, **75147**, 200126, 200193, 200306, 200307, 200308, 208740, 211251, 213201, **214110**, 223552, 223562, 228179, 228916, 228920, 228922, **234858**, 237550, 237804, 241221, 241265, 241267, 241282, 241283, 245062
SNNP	69088, 69178, 69326, 70161, 70187, 70636, 70795, 70874, 70891, 74649, **74651**, **74653**, 74656, 74663, 74665, 74666, 74670, 74681, 74685, 74686, 74687, 204622, 204626, 204631, 204633, 204636, 210903, 210906, 241706, 241708, 241709, 241715, 241720, 241721, 241722, **241723**, **241725**, 241728
Somali	70436, 70844, **70864**, 231179, 231199, 231201, **231204**, 231458
Tigray	19613, 19619, 19621, 19641, 31309, 71420, 71424, 71425, 71476, 71479, 71480, 71484, 71489, **71497**, 73799, 73802, 73805, 73955, 73963, 73964, 74061, **74101**, 74130, 74133, 74145, **74157**, 74168, 74177, 74181, 74183, 74191, 74203, 74220, 74222, 74225, 74231, **74933**, 207876, 220014, **234088**, 234112, 235468, 237300, 238388, 238391, 238392, 238394, 238396, 238397, 238403, 238405, 238408, 238425, 238428, **238445**, 238449, 238450, 242043, 243670
	**Improved varieties**
Bako ARC	Gemedi
Melkassa ARC	Geremew
Texas A&M University/ USA	**BTx623**

SNNP=Southern Nations, Nationalities, and Peoples', ARC=Agricultural Research Center
* Bold face entries were not genotyped

DNA extraction and DArT sequencing

Due to poor germination rate 313 sorghum genotypes were assayed for genomic analysis out of the 366 genotypes. Test genotypes were planted in a greenhouse at Holeta National Agricultural Biotechnology Research Center using seedling trays. Eight leaf disc samples were collected from

fresh and young leaf tissue using a 4×96 deep well sample collection plate three weeks after seedling emergence and sent to the Biosciences eastern and central Africa (BecA) hub of the International Livestock Research Institute (BecA-ILRI) in Kenya. Genomic DNA was extracted at BecA-ILRI/Kenya following the plant DNA extraction protocol for DArT [29]. The quality of DNA was checked for nucleic acid concentration and purity using a NanoDrop 2000 spectrophotometer (ND-2000 V3.5, NanoDrop Technologies, Inc). Samples were genotyped using the DArTseq protocol with 24,634 SNP markers. After eliminating the DArT loci with unknown chromosome positions and filtering markers with more than 10% missing data, a total of 11,611 markers distributed across the 10 chromosomes were maintained for analysis.

The markers used had SNPs with call rate > 97% and allele-calling equal or greater than 98% were selected. Genotypes with read depth less than the threshold were coded as missing. SNP markers have high rates of genotype missingness (>10%) and rare SNPs with <5% minor allele frequency (MAF) were discarded.

Marker-trait data analysis

Population structure

Principal component analysis (PCA) was computed in the R package ggplot2 [30]. Model-based maximum likelihood analysis of population structure was calculated using the ADMIXTURE program, a high-performance tool for estimation of ancestry in unrelated individuals [31]. To choose the optimum number of ancestral populations, ADMIXTURE was run with a 10-fold cross-validation procedure for K values varying from 2 to 20 and the K value with the lowest standard

error was selected. The graphical representation of the admixture patterns was depicted using the R package pophelper [32].

Marker–trait association

Data on anthracnose severity and GBS based on a total of 11,643 [illegible]ust SNP markers were used for GWAS analysis. The FarmCPU Model [33] was used to perform an association analysis using the R software's GAPIT package [16]. The model splits the mixed linear model into a fixed effect and a random effect model to minimize false negatives caused by confounding population structure, kinship, and SNPs. Kinship was calculated using the procedure described by Van Raden [34]. A coancestry matrix from ADMIXTURE was included as a covariate in GAPIT to reduce spurious associations.

The GWAS results were visually examined using the Manhattan and quantile-quantile (QQ) plots. The plots were generated using the CMPlot R package [35]. The cutoff of significant association was a False Discovery Rate (FDR) adjusted p-value less than 0.1 which was computed using the Benjamini and Hochberg procedure to control for multiple testing [36]. SNPs with high probability of contribution to important traits were tracked to the specific chromosome location based on the sorghum reference genome sequence, version 3.1.1 available at the Phytozome v12 [37].

Identification of candidate genes

Candidate genes were identified based on the significant SNP markers associated with the traits. This was achieved using the physical genome assembly of sorghum reference genome sequence, version 3.1.1 (https://phytozome.jgi.doe.gov/pz/portal.html#!info?alias=Org *Sbicolor*) serving as

identifying candidate genes between 20 kb on either side of significant SNPs. The putative functional candidate genes that co-localized with associated SNPs were annotated based on similarity to known annotated genes in other species such as Arabidopsis and tomato.

Results

Anthracnose resistance

Combined analysis of variance indicated significant differences (P < 0.01) due to the effects of genotype, season and genotype by season interaction for FAS and rAUDPC. These suggested the presence of considerable genetic variability among the test populations to select anthracnose resistant lines and aiding marker-trait analysis (Table 2).

Table 2. Mean squares and significant tests for final anthracnose severity (FAS) and relative area under disease progress curve (rAUDPC) amongst 366 sorghum lines assessed in three seasons (2016-2018) at Bako Agricultural Research Center in Ethiopia

		Parameters	
Source of variation	DF	FAS	rAUDPC
Seasons	2	1728**	27859**
Replication in season	2	59ns	5824**
Genotype	365	1402**	1620**
Genotype x season	730	251**	290**
Error	2194	51	71

DF=degree of freedom, FAS=Final anthracnose severity, rAUDPC= Relative area under disease progress curve, ** denote significant difference at P < 0.01and ns= non-significant

There were 32 genotypes that scored lower rAUDPC values at the final stage of severity rating that ranged between 29.4 (entry 74685) to 40.9 (223562). These lines were identified as moderately resistant making them ideal selections for anthracnose resistance breeding. Conversely, 334 genotypes were susceptible and expressed higher rAUDPC values ranging between 43.6 and 91.0. The rAUDPC showing the progression of disease severity on selected resistant and susceptible test

genotypes during severity assessment intervals is presented in Fig 2. Four genotypes (239182, 72564, 73048 and 238428) scored the highest rAUDPC values compared with the anthracnose susceptible check (Btx623). The mean values for final severity and rAUDPC are presented in Supplementary Table 1.

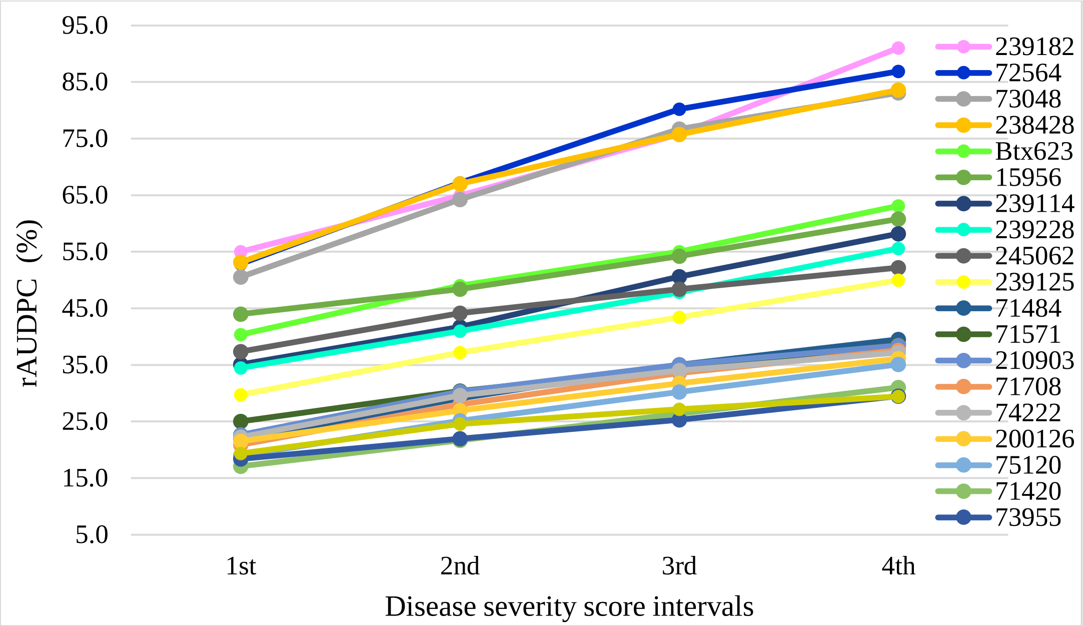

Figure 2. The relative area under disease progress curves (rAUDPC) of 10 anthracnose resistant and 10 susceptible sorghum genotypes including Btx623 among 366 genotypes evaluated in three seasons in Ethiopia.

Marker characterization

Population structure

Population structure analysis resolved eight sub-populations based on SNPs profiles. Cross-validation error estimates for the ADMIXTURE models steep decreased from K=2 to K=8, and increased steadily to a higher level at K=20 (Fig 3). The optimal number of sub-populations were at K = 8.

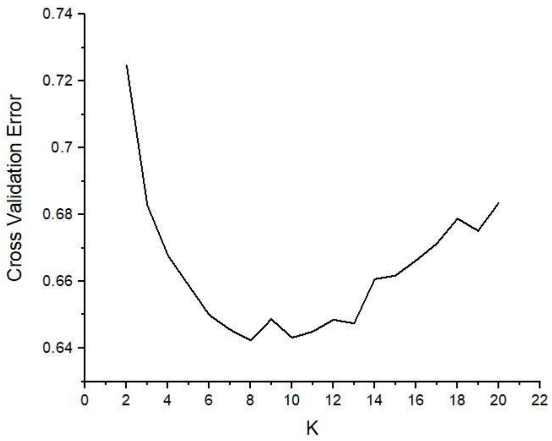

Figure 3. Plot depicting the cross-validation error rates values and K sub-sets varying from K=2 to K=20 based on ADMIXTURE analysis.

The ADMIXTURE analyses was performed for different K varying from 2 to 20 (Fig 4). Individual ancestry was estimated for different K-values. Each cluster consisted of genetically diverse and variable number of entries. About 65% of the assessed genotypes (203 accessions) were allocated in one of population that had high ancestry membership coefficient with a likelihood of more than 0.60. The remaining 35% of test populations represented accessions with high admixture which is expected from landrace populations. Population 7 represented larger number of entries collected from Oromiya, while Population 1 contained collections mainly from Afar region in Ethiopia.

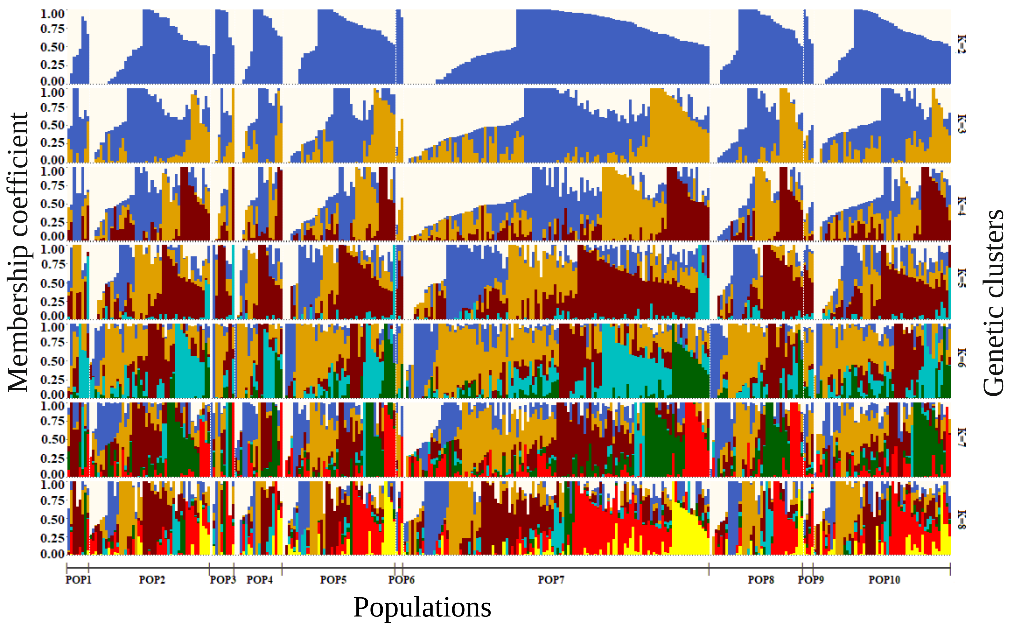

Figure 4. Population genetic structure among 313 Ethiopian sorghum genotypes. Each entry is represented as a single vertical line partitioned into segments corresponding to the inferred membership according to different K genetic clusters as indicated by the colors. Note: Pop1 to Pop10 represent collection area 1 to 10 resolved through GWAS.

A scree plot was generated to visualize the number of principal components. Overall 10 principal components were identified of which principal components 1 (PC1), PC2 and PC3 explained relatively the highest proportion to the total variance (Fig 5A). Principal coordinate analysis (PCA) using PC1 and PC2 stratified the test populations based on areas of collection (Fig 5B). The allocation of test genotypes were irrespective to origin of collections.

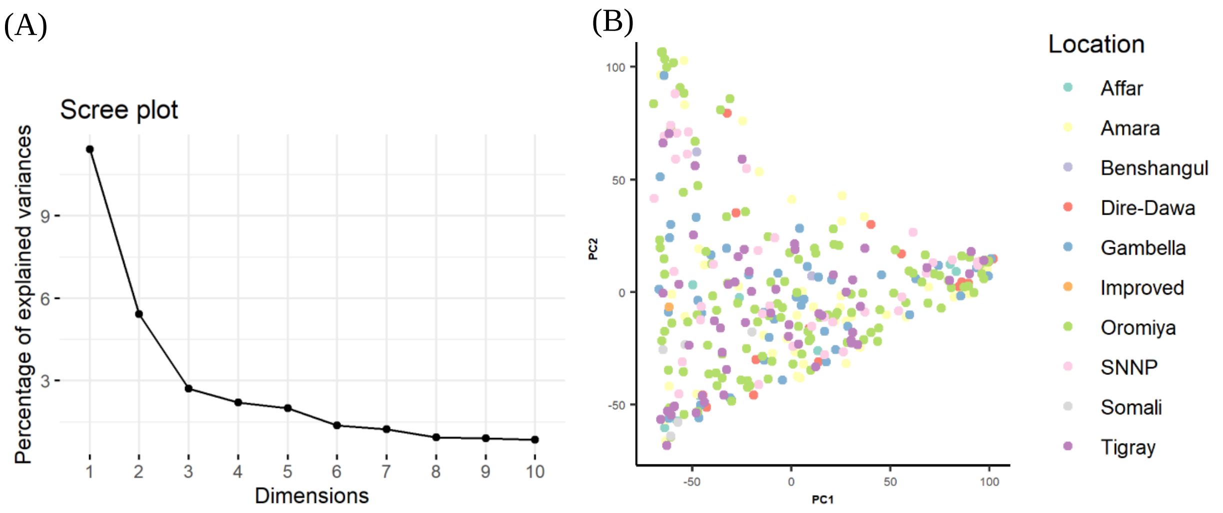

Figure 5. Principal component analysis among 313 sorghum collections based on 11,611 SNPs using the first two principal components. The large proportions of the variances contained in the data are retained by the first three principal components (A), while the relationship among collections between areas of origin is represented in (B).

Marker-trait association

A summary of the genome-wide association analysis of anthracnose resistance and SNP markers amongst 313 sorghum collections is presented in Table 3. A total of eight significant (P < 0.001) marker-trait associations were resolved on chromosomes 1, 4, 6, 8, 9 and 10 (Table 3 and Fig 6A). The quantile-quantile plots (Fig 6B) of p-values were examined to determine how well the models accounted for population structure and familial relatedness.

Three markers including markers *rs1887698* and *rs2681689* located on chromosome 9 and *rs1884746* on chromosome 10 had significant (*P* < 0.001) association with anthracnose resistance. Markers *rs1887698* and *rs2681689* significantly associated with chromosome 9 with P = 4.4×10^{-06} and 4.99×10^{-06}, respectively explaining 15.9% of the total variation for anthracnose resistance. Whereas marker *rs1884746* located on chromosome 10 (P = 4.28×10^{-05}) explained 15.0% of the total variation. Furthermore, SNPs significantly associated with anthracnose resistance were

detected on chromosomes 1, 8, 9, 6 and 4 which explained 4.86 to 5.23% of the total variation among the tested sorghum collections for anthracnose resistance. Overall, eight SNPs were found affecting anthracnose resistance (Table 3) of which four SNPs were above the cutoff point (Fig 6A). Hence, the significant MTAs localized on the specific chromosomes influence anthracnose resistance and could be exploited in gene introgression and selection. Also, the proportion of the phenotypic variation ($R^2 > 4$) observed for all significant markers suggest their possible influence on anthracnose resistance.

Table 3. Summary on genome-wide association analysis of anthracnose resistance and SNP markers amongst 313 sorghum collections indicating significant markers, alleles detected with chromosome number and position, significant value, coefficient of determination (R^2) and annotation.

Significant marker	Allele	Chromosome number	Position	P- value	R^2	Annotation
rs1887698	A/G	9	4.8E+07	4.40E-06	15.9	Sobic.009G126300 (Threonine-specific protein kinase)
rs2681689	C/T	9	815114	4.99E-06	15.9	Sobic.009G008800 (xylem cysteine proteases)
rs100028710	A/C	4	5.3E+07	1.59E-05	5.23	-
rs1938969	C/T	6	1561991	0.00068	5.21	-
rs1884746	A/G	10	1055302	4.28E-05	15.0	Sobic.010G012200 (Gluconokinase / Gluconate kinase)
rs5196058	C/T	9	4.8E+07	0.00074	4.95	-
rs2205151	T/G	8	5.4E+07	0.00032	4.87	-
rs100052771	A/G	1	2.2E+07	0.00032	4.86	-

- denote not available

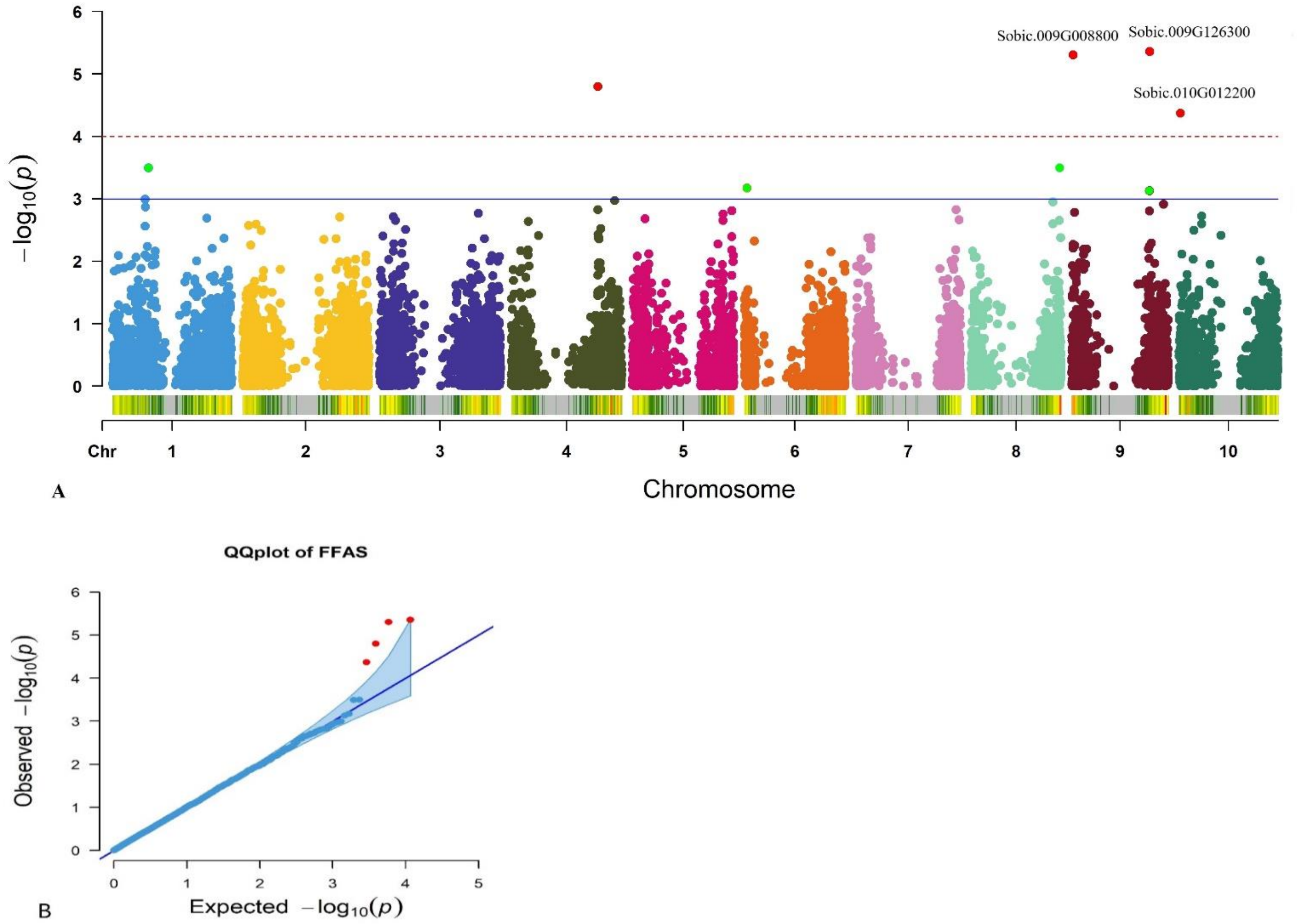

Figure 6. Genome-wide association of anthracnose resistance amongst 313 sorghum collections with 11,643 SNP markers using a FarmCPU Model: (a) Manhattan plots showing significant false discovery rate (FDR) adjusted P-value of ≤0.1 associated with anthracnose resistance and (b) Log Q-Q plots validating the FarmCPU Model and depicting consistency in reducing -log10(p-values) toward the expected level. A dash line represents the threshold from the FDR, and a blue line represents the significant threshold −Log10 (P) value.

Putative genes that condition anthracnose resistance

A blast search of potential anthracnose resistance genes identified three candidate genes. The two candidate genes were located on chromosome 9, while one gene on chromosome 10. The two genes on chromosome 9 are annotated with protein coding genes including *Sobic.009G008800* (*xylem cysteine proteases*) and *Sobic.009G126300 (Threonine-specific protein kinase)* linked to markers rs2681689 and rs1887698, respectively. In addition, one gene was annotated with protein

coding gene *Sobic.010G012200 (Gluconokinase/Gluconate kinase)* and linked to marker rs1884746 located on chromosome 10. The SNP data set used in study is presented in Supplementary Table 2.

Discussion

The existence of significant genotype by season interaction effects on anthracnose resistance suggests that there was considerable genetic diversity among the assessed genotypes and genotype performance varies across seasons. These results support earlier studies that there is potentially adequate genetic variation among Ethiopian sorghum to select for anthracnose resistance and to support genetic analysis, gene discovery and breeding efforts [8, 9, 11, 38].

Investigating the genetic bases of anthracnose resistance presents an opportunity for resistance breeding. To complement the ongoing need for anthracnose resistant sorghum genetic resources the current study explored the population structure and association of genomic regions with anthracnose resistance in a diverse population of sorghum collections from Ethiopia, its center of origin and diversity. Sorghum anthracnose resistance was confirmed through rigorous field screening in three seasons. Previous studies assessed the genetic basis of anthracnose resistance in sorghum [7]. The authors reported three loci on chromosome 5 amongst 335 US accessions using GWAS. In addition, [10] used 359 sorghum populations and identified quantitative trait loci on chromosome 8 conditioning resistance to anthracnose.

The population structure generated using hierarchical clustering, admixture, and principal component analysis identified a clear differentiation of the assessed sorghum collections (Figs 4, 5). The test population was grouped into eight distinct genetic groups (Fig 4).

Previous studies of population structure identified 11 genetic groups using 1,425 Ethiopian sorghum accessions [12]. In addition, a total of 318 Sudanese sorghum core collections were evaluated with 183,144 SNP markers using the model-based clustering method that portrayed five subpopulations [11]. Furthermore, 940 diverse sorghum landraces of Ethiopia were assessed using 54,080 SNP markers that identified 12 subpopulations [48].

Markers associated with anthracnose resistance were identified on chromosomes 9 and 10 suggesting the value of these genomic regions in gene introgression and pyramiding programs. Previously, markers for anthracnose resistance in sorghum collections from US were identified on chromosome 5 [7, 9] and chromosome 9 [8] using SNP markers. The new markers are potentially useful for marker assisted breeding of anthracnose resistance in the assessed sorghum populations [39].

The current study identified eight significant ($P < 0.001$) marker-trait associations on chromosomes 1, 4, 6, 8, 9 and 10 (Table 3 and Fig 6A). The quantile-quantile plots (Fig 6B) showed a perfect fit of the observed and expected population structures for anthracnose resistance using the FarmCPU Model. The novel genes are additions to previously identified genomic regions conditioning resistance to anthracnose, downy mildew, and head smut in sorghum [4,7-9]. Four SNP markers above the cutoff point (Fig 6A) are novel anthracnose resistance markers detected in this study. The markers are located on chromosomes 9, 10 and 4. Previously [4] used 242 mini core accessions and three sorghum cultivars and subjected to genome wide association analysis.

The authors reported anthracnose resistance gene on chromosome 8. In addition, [9] examined 114 recombinant inbred lines (RILs) obtained from crosses of sorghum anthracnose resistant line SC112-14 (PI533918) and susceptible line PI609251 and identified tightly linked anthracnose resistance locus on chromosome 5.

Through blast analysis the present study identified three candidate anthracnose resistance genes. The two genes are located on chromosome 9, while one gene on chromosome 10. The two genes located on chromosome 9 are annotated with protein coding genes *Sobic.009G008800* and *Sobic.009G126300* linked to markers rs1887698 and rs2681689, respectively. The *Sobic.009G008800* reportedly had strong association with annotated function xylem cysteine peptidase 1 of sorghum conditioning disease resistance. The biological effect of the gene includes programmed cell death, responsive to dehydration, while the annotated sorghum genes had roles in anthracnose resistance on chromosome 9 [40, 41]. The autho[illegible] reported that cysteine proteases is one of the first Arabidopsis proteases functioning in the immune system of sorghum. In a mutant strain of Arabidopsis, over expression of the aspartic protease Constitutive Disease Resistance 1 (CDR1) resulted in resistance to the virulent strain of *Pseudomonas syringae*, a bacterium that causes diseases of monocots, herbaceous dicots, and woody dicots. Cysteine proteases also contain a cysteine nucleophilic residue that performs a nucleophilic attack proteolysis resulting in an intermediate state where the enzyme is covalently attached to its substrate [42]. Cysteine proteases reportedly conferred immunity in tomato against diseases caused by *Phytophthora infestans* (Pinf) and *Cladosporium fulvum* (syn. Passalora fulva) [43, 44].

In the present study a second significant candidate gene, *Sobic.009G126300* was located on chromosome 9 linked to marker rs1887698 with annotated function similar to Serine/threonine-protein kinase CTR1. Serine/threonine-protein kinase is reportedly known for mediating drought stress tolerance in plants. Furthermore, threonine-protein kinase is believed to have a role in regulating ethylene hormone pathways conditioning drought response in senna (*Cassia angustifolia* Vahl.) [45]. The 3rd candidate gene identified in this study linked to the marker rs1884746 annotated with *Sobic.010G012200 (Gluconokinase/Gluconate kinase)*. Gluconate kinase is an essential enzyme of the oxidative pentose phosphate pathway which is responsible in NADPH supplies during fatty acid synthesis in developing embryos of *Thermotoga maritima* [46, 47].

The anthracnose resistance genes identified in the present study will be validated across multiple seasons and locations to serves as ideal molecular markers for anthracnose resistance breeding programs. The novel genes could be pyramided into anthracnose susceptible sorghum lines through marker-assisted selection. A combinations of QTLs could render durable resistance to sorghum anthracnose.

Conclusion

Marker trait association is key to identify genomic regions that are associated with anthracnose resistance for marker-assisted breeding in sorghum improvement programs. The present study identified four novel markers associated with anthracnose resistance designated as rs1887698, rs2681689, rs1884746 and rs100028710. The new genetic markers identified in the current sorghum populations are useful genomic resources for future parental selection, quantitative trait loci analysis, trait introgression, gene pyramiding and marker-assisted selection of anthracnose

resistance in sorghum breeding programs in Ethiopia or related agro-ecologies. Further studies are required to validate the significant markers identified in the present study.

Acknowledgments

The authors thank the National Research Foundation (NRF)/South Africa for financial support. The Integrated Genotyping Service and Support (IGSS) platform of the Biosciences Eastern and Central Africa (BecA-ILRI)/Kenya is acknowledged for genotyping the sorghum accessions.

Author Contributions

Conceptualization: GM HS DL.

Data curation: GM HS DL.

Formal analysis: GM HS EA DL.

Funding acquisition: GM HS DL.

Investigation: GM HS DL.

Methodology: GM HS EA DL.

Project administration: GM HS DL.

Resources: GM HS EA DL.

Writing - original draft: GM HS EA.

Writing - review & editing: GM HS EA DL.

References

1. Dahlberg J, Berenji J, Sikora V, Latkovic D. Assessing sorghum [*Sorghum bicolor* (L) Moench] germplasm for new traits: food, fuels and unique uses. Maydica. 2011; 56:85–92.
2. Nida H, Girma G, Mekonen M, Lee S, Seyoum A, Dessalegn K, et al. Identification of sorghum grain mold resistance loci through genome wide association mapping. Journal of Cereal Science. 2019; 85:295–304.
3. Ejeta G, Gressel J. Integrating new technologies for striga control: towards ending the witch-hunt: World Scientific Publishing; 2007. 345pp.
4. Ahn E, Hu Z, Perumal R, Prom LK, Odvody G, Upadhyaya HD, et al. Genome wide association analysis of sorghum mini core lines regarding anthracnose, downy mildew, and head smut. PLOS ONE. 2019; 14:e0216671.
5. Chala A, Brurberg MB, Tronsmo AM. Prevalence and intensity of sorghum anthracnose in Ethiopia. Journal of SAT Agricultural Research. 2007; 5:145–298.
6. Thakur RP, Mathur K. Anthracnose. In: Compendium of sorghum diseases. (Eds. R. A. Frederiksen and G. N. Odvody). The American Phytopathological Society, St Paul, MN, USA; 2000. pp:10–12.
7. Cuevas HE, Prom LK, Cooper EA, Knoll JE, Ni X. Genome- wide association mapping of anthracnose (*Colletotrichum sublineolum*) resistance in the US sorghum association panel. The Plant Genome. 2018; 11:170099. https://doi.org/10.3835/plantgenome2017.11.0099
8. Cuevas HE, Prom LK, Cruet-Burgos CM. Genome-wide association mapping of anthracnose (*Colletotrichum sublineolum*) resistance in NPGS Ethiopian sorghum germplasm. G3: Genes, Genomes, Genetics. 2019; 9:2879–2885. https://doi.org/10.1534/g3.119.400350
9. Cruet-Burgos CM, Cuevas HE, Prom LK, Knoll JE, Stutts LR, Vermerris W. Genomic dissection of anthracnose (*Colletotrichum sublineolum*) resistance response in sorghum differential line SC112-14. G3: Genes, Genomes, Genetics. 2020; 10:1403–1412. https://doi.org/10.1534/g3.120.401121

10. Prom LK, Ahn E, Isakeit T, Magill C. GWAS analysis of sorghum association panel lines identifies SNPs associated with disease response to Texas isolates of *Colletotrichum sublineola*. Theoretical and Applied Genetics. 2019; 132:1389–1396. https://doi.org/10.1007/s00122-019-03285

11. Cuevas HE, Prom LK. Evaluation of genetic diversity, agronomic traits, and anthracnose resistance in the NPGS Sudan sorghum core collection. BMC Genomics. 2020; 21:88. https://doi.org/10.1186/s12864-020-6489-0

12. Girma G, Nida H, Seyoum A, Mekonen M, Nega A, Lule D, et al. A large-scale genome-wide association analyses of Ethiopian sorghum landrace collection reveal loci associated with important traits. Frontiers in Plant Science. 2019; 10:691. https://doi.org/10.3389/fpls.2019.00691

13. Rhodes DH, Hoffmann Jr L, Rooney WL, Ramu P, Morris GP, Kresovich S. Genome-wide association study of grain polyphenol concentrations in global sorghum [*Sorghum bicolor* (L.) Moench] germplasm. Journal of Agricultural and Food Chemistry. 2014; 62: 10916–10927. https://doi.org/10.1021/jf503651t

14. Batley J, Edwards D. SNP applications in plants. Association mapping in plants: Springer; 2007. pp:95-102.

15. Chen J, Chopra R, Hayes C, Morris G, Marla S, Burke J, et al. Genome‐ wide association study of developing leaves' heat tolerance during vegetative growth stages in a sorghum association panel. The Plant Genome. 2017; 10(2):1–15. https://doi.org/10.3835/plantgenome2016.09.0091

16. Lipka AE, Tian F, Wang Q, Peiffer J, Li M, Bradbury PJ, et al. GAPIT: Genome association and prediction integrated tool. Bioinformatics. 2012; 28(18):2397-2399. https://doi:10.1093/bioinformatics/bts444

17. Vavilov NI. The origin, variation, immunity and breeding of cultivated plants. Translated by Chester K.S. Ronald Press, New York; 1951. 366pp.

18. Doggett H. Sorghum. Longman Scientific and Technical, London; 1988.

19. Ayana A, Bekele E. Geographical patterns of morphological variation in sorghum (*Sorghum bicolor* (L.) Moench) germplasm from Ethiopia and Eritrea: quantitative characters. Euphytica. 2000; 115:91–104.

20. Smith CW, Frederiksen RA. Sorghum: origin, history, technology, and production: John Wiley and Sons, USA; 2000. pp.840.

21. Paterson AH, Bowers JE, Bruggmann R, Dubchak I, Grimwood J, Gundlach H, et al. The *Sorghum bicolor* genome and the diversification of grasses. Nature. 2009; 457:551–556.

22. Patil NY, Klein RR, Williams CL, Collins SD, Knoll JE, Burrell AM, et al. Quantitative trait loci associated with anthracnose resistance in sorghum. Crop Science. 2017; 57: 877–890.

23. Rhodes DH, Hoffmann L, Rooney WL, Herald TJ, Bean S, Boyles R, et al. Genetic architecture of kernel composition in global sorghum germplasm. BMC Genomics. 2017; 18:1–8. https://doi.org/10.1186/s12864-016-3403-x

24. Prom LK, Perumal R, Erpelding JE, Isakeit T, Montes-Garcia N, Magill CW. A pictorial technique for mass screening of sorghum germplasm for anthracnose (*Colletotrichum sublineolum*) resistance. The Open Agriculture Journal. 2009; 3:20–25.

25. Chala A, Tronsmo AM. Evaluation of Ethiopian sorghum accessions for resistance against *Colletotrichum sublineolum*. European Journal of Plant Pathology. 2012; 132:179–189.

26. Campbell CL, Madden LV. Introduction to Plant Disease Epidemiology: John Wiley and Sons; 1990. pp.560.

27. Matschegewski C, Zetzsche H, Hasan Y, Leibeguth L, Briggs W, Ordon F. Genetic variation of temperature-regulated curd induction in cauliflower: elucidation of floral transition by genome-wide association mapping and gene expression analysis. Frontiers in Plant Science. 2015; 6:1–14. https://doi.org/10.3389/fpls.2015.00720

28. SAS. Statistical analysis system, version 9.0. Cary, NC: SAS Institute Inc; 2012.

29. DArT. Extraction protocol for DArT. Camberra, Australia: Diversity Arrays Technology; 2014.

30. Wickham H. Elegant graphics for data analysis (ggplot2). Applied Spatial Data Analysis R; 2009.

31. Alexander DH, Lange K. Enhancements to the ADMIXTURE algorithm for individual ancestry estimation. BMC Bioinformatics. 2011; 12:1–6. https://doi.org/10.1186/1471-2105-12-246

32. Francis RM. Pophelper: an R package and web app to analyse and visualize population structure. Molecular Ecology Resources. 2017; 17:27–32. https://doi.org/10.1111/1755-0998.12509

33. Liu X, Huang M, Fan B, Buckler ES, Zhang Z. Iterative usage of fixed and random effect models for powerful and efficient genome-wide association studies. PLOS Genetics. 2016; 12:e1005767. https://doi.org/10.1371/journal.pgen.1005767

34. VanRaden PM. Efficient methods to compute genomic predictions. Journal of Dairy Science. 2008; 91:4414–4423.

35. Zhao X, Han Y, Li Y, Liu D, Sun M, Zhao Y, et al. Loci and candidate gene identification for resistance to *Sclerotinia sclerotiorum* in soybean (*Glycine max* L. Merr.) via association and linkage maps. The Plant Journal. 2015; 82:245–255. https://doi.org/10.1111/tpj.12810

36. Benjamini Y, Hochberg Y. Controlling the false discovery rate: a practical and powerful approach to multiple testing. Journal of the Royal Statistical Society: series B (Methodological). 1995; 57:289–300.

37. McCormick RF, Truong SK, Sreedasyam A, Jenkins J, Shu S, Sims D, et al. The sorghum bicolor reference genome: improved assembly, gene annotations, a transcriptome atlas, and signatures of genome organization. The Plant Journal. 2018; 93:338–354.

38. Cuevas HE, Louis K. Prom, Elizabeth A. Cooper, Joseph E. Knoll a, Ni X. Genome-wide association mapping of anthracnose (*Colletotrichum sublineolum*) resistance in the U.S. sorghum association panel. The Plant Genome. 2018; 11:13.

39. Kamfwa K, Cichy KA, Kelly JD. Genome-wide association study of agronomic traits in common bean. The Plant Genome. 2015; 8(2):1–12.

40. Pogány M, Dankó T, Kámán-Tóth E, Schwarczinger I, Bozsó Z. Regulatory proteolysis in Arabidopsis-pathogen interactions. International Journal of Molecular Sciences. 2015; 16: 23177–23194. https://doi.org/10.3390/ijms161023177 PMID: 26404238

41. Patil NY. Investigating the genetic basis of agronomically important traits in sorghum. A Dissertation; 2016. pp.140.

42. Van der Hoorn RA, Leeuwenburgh MA, Bogyo M, Joosten MH, Peck SC. Activity profiling of papain-like cysteine proteases in plants. Plant Physiology. 2004; 135:1170–1178. https://doi.org/10.1104/pp.104.041467

43. Shindo T, Misas-Villamil JC, Hörger AC, Song J, van der Hoorn RA. A role in immunity for Arabidopsis cysteine protease RD21, the ortholog of the tomato immune protease C14. PLOS ONE. 2012; 7:e29317. https://doi.org/10.1371/journal.pone.0029317

44. van Esse HP, Van't Klooster JW, Bolton MD, Yadeta KA, Van Baarlen P, Boeren S, et al. The *Cladosporium fulvum* virulence protein Avr2 inhibits host proteases required for basal defense. The Plant Cell. 2008; 20:1948–1963. https://doi.org/10.1105/tpc.108.059394

45. Mehta RH, Ponnuchamy M, Kumar J, Reddy NRR. Exploring drought stress-regulated genes in senna (*Cassia angustifolia* Vahl.): a transcriptomic approach. Functional and Integrative Genomics. 2017; 17:1–25. https://doi.org/10.1007/s10142-016-0523-y

46. Zhang Y, Zagnitko O, Rodionova I, Osterman A, Godzik A. The FGGY carbohydrate kinase family: insights into the evolution of functional specificities. PLOS Computational Biology. 2011; 7:e1002318. https://doi.org/10.1371/journal.pcbi.1002318

47. Dos Santos Fernandes L, Correa FM, Ingram KT, de Almeida A-AF, Royaert S. QTL mapping and identification of SNP-haplotypes affecting yield components of *Theobroma cacao* L. Horticulture Research. 2020; 7:1–18.

48. Menamo T, Kassahun B, Borrell A, Jordan D, Tao Y, Hunt C, Mace E. Genetic diversity of Ethiopian sorghum reveals signatures of climatic adaptation. Theoretical and Applied Genetics. 2021; 134: 731–742.

Click here to access/download

Supporting Information

Supplemental Table 2 SNP data set PONE-D-21-10117.csv

Click here to access/download
Supporting Information
Supplemental Table 1 Mean FAS and rAUDPC values.csv

Revised Manuscript

Genome-wide association analysis of anthracnose resistance in sorghum [*Sorghum bicolor* (L.) Moench]

Girma Mengistu[1,2]*, Hussein Shimelis[1], Ermias Assefa[3] and Dagnachew Lule[2]

1 University of KwaZulu-Natal, School of Agricultural, Earth and Environmental Sciences, Private Bag X01, Scottsville 3209, Pietermaritzburg, South Africa, 2 Oromia Agricultural Research Institute, P.O. Box 81265, Addis Ababa, Ethiopia, 3 Ethiopian Biotechnology Institute, P.O. Box 5954, Addis Ababa, Ethiopia

* germame2004@gmail.com

Abstract

In warm-humid ago-ecologies of the world, sorghum [*Sorghum bicolor* (L.) Moench] production is severely affected by anthracnose disease caused by *Colletotrichum sublineolum* Henn. New sources of anthracnose resistance should be identified to introgress novel genes into susceptible varieties in resistance breeding programs. The objective of this study was to determine genome wide association of Diversity Arrays Technology Sequencing (DArTseq) based single nucleotide polymorphisms (SNP) markers and anthracnose resistance genes in diverse sorghum population for resistance breeding. Three hundred sixty-six sorghum populations were assessed for anthracnose resistance in three seasons in western Ethiopia using artificial inoculation. Data on anthracnose severity and the relative area under disease progress curve were computed. Furthermore, the test populations were genotyped using SNP markers with DArTseq protocol. Population structure analysis and genome-wide association mapping were undertaken based on 11,643 SNPs with <10% missing data. The evaluated population was grouped into ~~ten~~ eight distinct genetic clusters. A total of eight significant (P < 0.001) marker-trait associations (MTAs) were detected explaining 4.86-15.9% of the phenotypic variation for anthracnose resistance~~.~~ out of which ~~Amongst the eight SNPs that were significantly associated with anthracnose resistance~~ four were above the cutoff point. The significant MTAs present in the assessed sorghum population are useful for marker-assisted selection (MAS) ~~by~~ in anthracnose resistance breeding programs and for gene and quantitative trait loci (QTL) mapping.

Keywords: anthracnose resistance, DArTseq technology, Ethiopia, marker-trait association, quantitative trait loci, sorghum anthracnoseQTL

Introduction

Sorghum [*Sorghum bicolor* (L.) Moench, 2n=2x=20] is an important cereal crop cultivated globally for multiple uses [1]. It is a powerhouse mainstay crop in arid and semi-arid agro-ecologies globally due to its relatively higher drought tolerance compared with other common cereal crops such as maize and wheat. Sorghum production and productivity is affected by various constraints notably by biotic stresses such as diseases, weeds (*Striga* species) and insect pests. Anthracnose, grain mold, leaf blight, rust and smut are the most important diseases of sorghum, while stem borer, shoot fly, termites and birds are the common pests attacking the crop [2-4].

Sorghum anthracnose caused by the fungal pathogen *Colletotrichum sublineolum* Henn. (previously known as *C. graminicola* [Ces.] G.W. Wilson) is amongst the most important diseases of the crop. Anthracnose causes yield and quality losses in sorghum production. For instance, grain yield loss of 50-70% have been reported in susceptible sorghum varieties in the drier and humid agro-ecologies of Ethiopia [5, 6]. Development and deployment of anthracnose resistant sorghum varieties is an economic and environmentally friendly approach that could bolster sustainable sorghum production and productivity. Understanding the genetic basis and dissection of genes conditioning anthracnose resistance using genomic tools enhances gene introgression and marker-assisted selection [4, 7-10].

Genome-wide association study (GWAS) has been widely used to resolve complex genes controlling economic traits in crop plants including anthracnose resistance for effective breeding

and genetic analysis [4, 7, 8, 11]. Genome-wide genetic analysis enables to delineate genomic regions such as markers, genes or quantitative trait loci (QTL) associated with key component traits for marker-assisted breeding, gene discovery or gene introgression [7, 9]. Genome wide association analysis is dependent on marker-trait association (MTA) using representative markers and genetically diverse test populations which may include landraces, advanced breeding lines and improved cultivars [12, 13]. Identification of marker to trait associations will enhance gene introgression and selection gains through marker-assisted breeding.

Single nucleotide polymorphisms (SNP) is ~~are~~ a marker of choice for genetic diversity analysis, characterization of genetic resources, cultivar identification, heterotic grouping, construction of high-resolution genetic maps, linkage disequilibrium-based association mapping and genetic diagnostics [14]. SNPs have been used in GWAS of various traits in sorghum including genetic analysis for resistance to diseases such as anthracnose [4, 7-9], grain mold [2], downy mildew, and head smut [4] and tolerance to drought [15]. Diversity Arrays Technology Sequencing (DArTseq) based single nucleotide polymorphisms (SNPs) is a powerful genomic tool used in GWAS of diverse agriculturally important crop traits [16].

Ethiopia is the center of origin and diversity of both cultivated and wild sorghum [17-20]. The country maintains more than 9000 sorghum accessions through the Ethiopian Biodiversity Institute (EBI) [12]. Sorghum genetic resources from Ethiopia have been used globally in genetic analysis and cultivar development programs [2, 12]. Some novel genes conditioning resistance to grain mold [2] and anthracnose [8] were reported amongst Ethiopian sorghum. The sorghum genetic

resources maintained by the EBI should be systematically explored and used in improvement programs.

The sorghum genome was previously sequenced as a foundational database to guide genetic analysis and breeding [21]. Also Furthermore, various studies reported sorghum genomic regions associated with genes governing the inheritance of different traits such as anthracnose resistance [4, 7, 9, 10, 22], male sterility [12], flowering and plant height [12], heat tolerance [15] and grain polyphenol content [13]. Quantitative trait loci (QTL) conditioning the contents of protein, fat, and starch were reported in sorghum [23]. There are limited genomic studies that examined marker-trait associations including for anthracnose resistance with diverse and representative genetic pool of sorghum in Ethiopia.

The Bako areas in western parts of Ethiopia are known to maize and sorghum production. The areas are a known hotspot sites for sorghum anthracnose owing to its warm temperatures and high relative humidity [5]. To initiate anthracnose resistance breeding program a large number of sorghum genetic resources initially collected by the Ethiopian Biodiversity Institute (EBI) from nine regions in Ethiopia, were provided by the Melkassa Agricultural Research Center. were assembled by the Bako Agricultural Research Center in Ethiopia. The genetic resources preserved by the Bako Agricultural Research Center should be further profiled using high density SNP markers and anthracnose resistance to identify and introgress novel genes into susceptible varieties by resistance breeding programs to enhance productivity of the crop. In light of the above background, the objective of this study was to determine genome wide association of Diversity

Arrays Technology Sequencing based single nucleotide polymorphisms markers and anthracnose resistance genes in diverse sorghum population of Ethiopia for resistance breeding.

Materials and methods

Plant materials

The study used a population of 366 sorghum collections sourced from different geographical locations in Ethiopia including the Afar, Amhara, Benshangul Gumuz, Dire Dawa, Gambella, Oromiya, SNNP, Somali, and Tigray regions acquired from Melkassa Agricultural Research Center in Ethiopia. Three improved varieties ‘Gemedi’, ‘Geremew’ and ‘Btx623’ were included as comparative controls. Btx623 is anthracnose susceptible variety obtained from Texas A and& M University, USA. The descriptions of the test accessions are presented in Table 1.

Study sites and experimental design

The genotypes were evaluated for anthracnose resistance at Bako (9º6’ N; 37º9’ E) Agricultural Research Center (BARC) in Ethiopia. The center receives annual rainfall of 1,600 mm, while the mean maximum and minimum temperatures are 29 ºC and 13 ºC, respectively. The mean monthly relative humidity varies from 46 to 57%. The main rainy season ranges from May to October, with the most rain received in July and August. Short rains are also received during March to June. The trends of weather data for BARC during the study periods (2016-2018) are summarized in Fig 1. The soil type of the study site is nitosol. The genotypes were established using a 61 x 6 row by column incomplete block design with three replications. Each plot consisted of a single row of 2.1 m long with inter-row and intra-row spacing of 75 cm and 15 cm, respectively.

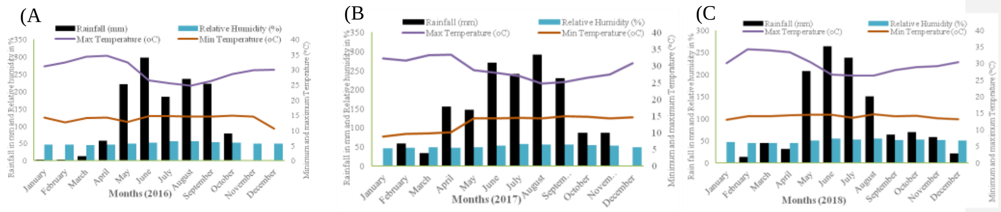

Figure 1. Rainfall, relative humidity, and minimum and maximum temperatures of Bako Agricultural Research Center in 2016 (A), 2017 (B) and 2018 (C). (Source: Bako Agricultural Research Center/Ethiopia)

Pathotyping for anthracnose reaction, data collection and analysis

Based on preliminary evaluations, single virulent anthracnose spore sourced from the Bako area was aseptically isolated, multiplied and used for inoculation of plants 45 days after sowing using the commonly used procedure ~~of~~ [24]. The 366 genotypes were rigorously screened for anthracnose resistance at Bako for three seasons (2016–2018). Data on percentage severity of leaf area damaged by the anthracnose were recorded beginning from 15 days after inoculation five times at 10 days' intervals from five randomly tagged plants. The percentage of total leaf area of plants damaged by anthracnose were recorded following [25]. The final disease rating was measured 55 days after inoculation and was referred to as a final anthracnose severity (FAS). FAS was used to distinguish anthracnose reaction of lines based on the level of severity. Mean severity percentage values for each plot were used for data analysis.

Disease severity for anthracnose was used to calculate the area under disease progress curve (AUDPC). The AUDPC were calculated for each sorghum accession based on [26] as follows:

$$\mathrm{AUDPC} = \sum_{i=1}^{n-1} [(X_i + X_{i+1})/2]\,(t_{i+1} - t_i)$$

The final disease rating was measured 55 days after inoculation and was referred to as a final anthracnose severity (FAS). The AUDPC values were converted into relative area under disease

progress curve (rAUDPC) as a ratio of the actual AUDPC of a sorghum entry to the AUDPC of a susceptible landrace (Acc#239182) across the three cropping seasons. The data were checked for normality, homogeneity of variance and validity for analysis of variance following the Bartlet (1974) test [27]. Data collected were analyzed using SAS computer software (SAS, 2012 [28].

Table 1. List of 366 sorghum genotypes used in this study

**Collection regions or research centres**	**Accessions**
	Landraces
Afar	72564,72998, **73003**, 73006, 73007, **73008**, **73019**, **73026,** 73643, 73645, 206210, 206212
Amhara	69252, **70376**, 72443, 72467, **72474**, 72477, **72520**, **72524**, 72526, 72616, 73037, 73041, 73042, 73045, 73048, 73049, 73074, **73079**, **73095**, 75274, 75455, 200539, 210945, 210949, 210971, 210974, **211237**, **212640**, 213354, 214845, 214852, 226047, 226048, 226054, 226057, 228112, **228115**, 229887, 239154, 239179, 239180, 239182, **239184**, 239187, 239188, 239194, 239197, 239219, 239228, 239250, 242052, 243645, 243650, 243657
Benshangul	SC283-14, **ETSL100375**, **PML981475**, 229832
Dire Dawa	70742, 71161, 71180, 228840, 239114, **239115**, 239116, 239117, 239118, 239119, 239123, 239124, 239125, 239126, **239127**, 239129, **239131**, 239132, 239133, 239134, **239135**, 239137
Gambella	69372, 69412, 70027, 70028, 70051, 71569, 71570, 71571, 71574, 71623, 71624, 71625, 71628, 71631, 71635, 71642, 71643, 71644, 71648, 71653, 71654, 71656, 71658, 71698, **71700**, 71701, 71708, 71711, 71712, 71714, 71720, 74914, 200522, 201433, 206149, 206154, 211209, 211210, **222885**
Oromiya	9110, **9116**, 15830, **15832**, **15877**, 15890, 15897, 15904, **15908**, 15914, 15932, 15935, 15956, **16113**, 16116, 16133, **16135**, 16152, 16162, **16163**, 16168, 16171, 16173, 16176, 16177, 16180, 16206, 16208, 16212, 16213, 16440, 16450, 16451, 16477, 16487, 16489, 17518, 69534, **69540**, 69553, 70282, 70471, 70704, 70842, 70859, 70943, 70967, 70998, 71044, 71110, 71137, 71154, 71165, 71168, 71169, 71177, 71194, 71319, 71334, **71337**, 71363, 71372, 71374, 71392, **71395**, 71466, 71500, 71502, 71503, **71507**, 71513, 71516, 71524, **71544**, 71545, 71546, 71547, 71548, 71549, **71550**, 71551, 71553, 71555, 71556, 71557, 71558, 71559, 71560, 71562, **71563**, 75003, 75004, **75006**, 75114, 75115, **75118**, 75119, 75120, 75123, 75132, 75143, 75146, **75147**, 200126, 200193, 200306, 200307, 200308, 208740, 211251, 213201, **214110**, 223552, 223562, 228179, 228916, 228920, 228922, **234858**, 237550, 237804, 241221, 241265, 241267, 241282, 241283, 245062
SNNP	69088, 69178, 69326, 70161, 70187, 70636, 70795, 70874, 70891, 74649, **74651**, **74653**, 74656, 74663, 74665, 74666, 74670, 74681, 74685, 74686, 74687, 204622, 204626, 204631, 204633, 204636, 210903, 210906, 241706, 241708, 241709, 241715, 241720, 241721, 241722, **241723**, **241725**, 241728
Somali	70436, 70844, **70864**, 231179, 231199, 231201, **231204**, 231458
Tigray	19613, 19619, 19621, 19641, 31309, 71420, 71424, 71425, 71476, 71479, 71480, 71484, 71489, **71497**, 73799, 73802, 73805, 73955, 73963, 73964, 74061, **74101**, 74130, 74133, 74145, **74157**, 74168, 74177, 74181, 74183, 74191, 74203, 74220, 74222, 74225, 74231, **74933**, 207876, 220014, **234088**, 234112, 235468, 237300, 238388, 238391, 238392, 238394, 238396, 238397, 238403, 238405, 238408, 238425, 238428, **238445**, 238449, 238450, 242043, 243670
	**Improved varieties**
Bako ARC	Gemedi
Melkassa ARC	Geremew
Texas A&M University/ USA	**BTx623**

SNNP=Southern Nations, Nationalities, and Peoples', ARC=Agricultural Research Center
* Bold face entries were not genotyped

DNA extraction and DArT sequencing

Due to poor germination rate 313 sorghum genotypes were assayed for genomic analysis out of the 366 entriesgenotypes. Test genotypes were planted in a greenhouse at Holeta National Agricultural Biotechnology Research Center using seedling trays. Eight leaf disc samples were

collected from fresh and young leaf tissue using a 4×96 deep well sample collection plate three weeks after seedling emergence and sent to the Biosciences eastern and central Africa (BecA) hub of the International Livestock Research Institute (BecA-ILRI) in Kenya. Genomic DNA was extracted at BecA-ILRI/Kenya following the plant DNA extraction protocol for DArT [29]. The quality of DNA was checked for nucleic acid concentration and purity using a NanoDrop 2000 spectrophotometer (ND-2000 V3.5, NanoDrop Technologies, Inc). Samples were genotyped using the DArTseq protocol with 24,634 SNP markers. After eliminating the DArT loci with unknown chromosome positions and filtering markers with more than 10% missing data, a total of 11,611 markers distributed across the 10 chromosomes were maintained for analysis.

The markers used had SNPs with call rate > 97% and allele-calling equal or greater than 98% were selected. Genotypes with read depth less than the threshold were coded as missing. SNP markers have high rates of genotype missingness (>10%) and rare SNPs with <5% minor allele frequency (MAF) were discarded.

Marker-trait data analysis

Population structure

Principal component analysis (PCA) was computed in the R package ggplot2 [30]. Model-based maximum likelihood analysis of population structure was calculated using the ADMIXTURE program, a high-performance tool for estimation of ancestry in unrelated individuals [31]. To choose the optimum number of ancestral populations, ADMIXTURE was run with a 10-fold cross-validation procedure for K values varying from 2 to 20 and the K value with the lowest standard

error was selected. The graphical representation of the admixture patterns was depicted using the R package pophelper [32].

Marker–trait association

Data on anthracnose severity and GBS based on a total of 11,643 robust SNP markers were used for GWAS analysis. The FarmCPU Model [33] was used to perform an association analysis using the R software's GAPIT package [16]. The model splits the mixed linear model into a fixed effect and a random effect model to minimize false negatives caused by confounding population structure, kinship, and SNPs. Kinship was calculated using the procedure described by Van Raden [34]. A coancestry matrix from ADMIXTURE was included as a covariate in GAPIT to reduce spurious associations.

The GWAS results were visually examined using the Manhattan and quantile-quantile (QQ) plots. The plots were generated using the CMPlot R package [35]. The cutoff of significant association was a False Discovery Rate (FDR) adjusted p-value less than 0.1 which was computed using the Benjamini and Hochberg procedure to control for multiple testing [36]. SNPs with high probability of contribution to important traits were tracked to the specific chromosome location based on the sorghum reference genome sequence, version 3.1.1 available at the Phytozome v12 [37].

Identification of candidate genes

Candidate genes were identified based on the significant SNP markers associated with the traits. This was achieved using the physical genome assembly of sorghum reference genome sequence, version 3.1.1 (https://phytozome.jgi.doe.gov/pz/portal.html#!info?alias=Org *Sbicolor*) serving as

Formatted: Left, Space After: 8 pt, Line spacing: 1.5 lines

identifying candidate genes between 20 kb on either side of significant SNPs. The putative functional candidate genes that co-localized with associated SNPs were annotated based on similarity to known annotated genes in other species such as Arabidopsis and tomato.

Results

Anthracnose resistance

Combined analysis of variance indicated significant differences (P < 0.01) due to the effects of genotype, season and genotype x by season interaction for FAS and rAUDPC. These suggested the presence of considerable genetic variability among the test populations to select anthracnose resistant lines and aiding marker-trait analysis (Table 2).

Table 2. Mean squares and significant tests for final anthracnose severity (FAS) and relative area under disease progress curve (rAUDPC) amongst 366 sorghum lines assessed in three seasons (2016-2018) at Bako Agricultural Research Center in Ethiopia

		Parameters	
Source of variation	DF	FAS	rAUDPC
Seasons	2	1728**	27859**
Replication in season	2	59ns	5824**
Genotype	365	1402**	1620**
Genotype x season	730	251**	290**
Error	2194	51	71

DF=degree of freedom, FAS=Final anthracnose severity, rAUDPC= Relative area under disease progress curve, ** denote significant difference at P < 0.01and ns= non-significant

There were 32 genotypes that scored lower rAUDPC values at the final stage of severity rating that ranged between 29.4 (entry 74685) to 40.9 (223562). These lines were identified as moderately resistant making them ideal selections for anthracnose resistance breeding. Conversely, 334 genotypes were susceptible and expressed higher rAUDPC values ranging between 43.6 and 91.0. The rAUDPC showing the progression of disease severity on selected resistant and susceptible test

genotypes during severity assessment intervals is presented in Fig 2. Four genotypes (239182, 72564, 73048 and 238428) scored the highest rAUDPC values compared with the anthracnose susceptible check (Btx623). -The mean values for final severity and rAUDPC are presented in Supplementary Table 1.

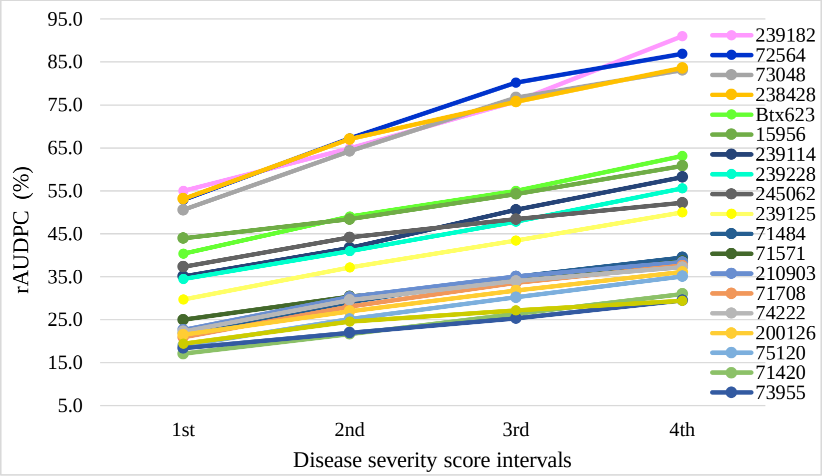

Figure 2. The rRelative area under disease progress curves (The rAUDPC)) of 10 anthracnose resistant and 10 susceptible sorghum genotypes that included a susceptible check (including Btx623) among 366 genotypes evaluated in three seasons in Ethiopia.

Marker characterization

Population structure

Population structure analysis resolved eight sub-populations based on SNPs profiles. Cross-validation error estimates for the ADMIXTURE models steep decreased from K=2 to K=8, and increased steadily to a higher level at K=20 (Fig 3). The optimal number of sub-populations were at K = 8.

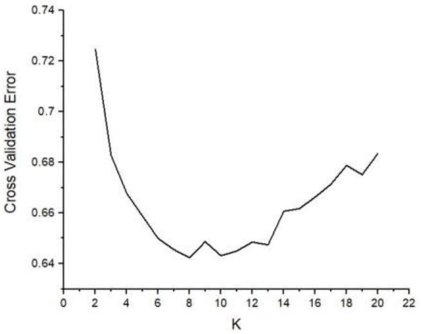

Figure 3. Plot depicting the cross-validation error rates values and K sub-sets varying from K=2 to K=20 based on ADMIXTURE analysis.

The ADMIXTURE analyses was performed for different K varying from 2 to 20~~identified 10 populations amongst the assessed sorghum germplasm~~ (Fig 4). Individual ancestry was estimated for different K-values. Each cluster consisted of genetically diverse and variable number of entries. About 65% of the assessed genotypes (203 accessions) were allocated in one of population that had high ancestry membership coefficient with a likelihood of more than 0.60. The remaining 35% of test populations represented accessions with high admixture which is expected from landrace populations. Population 7 represented larger number of entries collected from Oromiya, while Population 1 contained collections mainly from Afar region in Ethiopia.

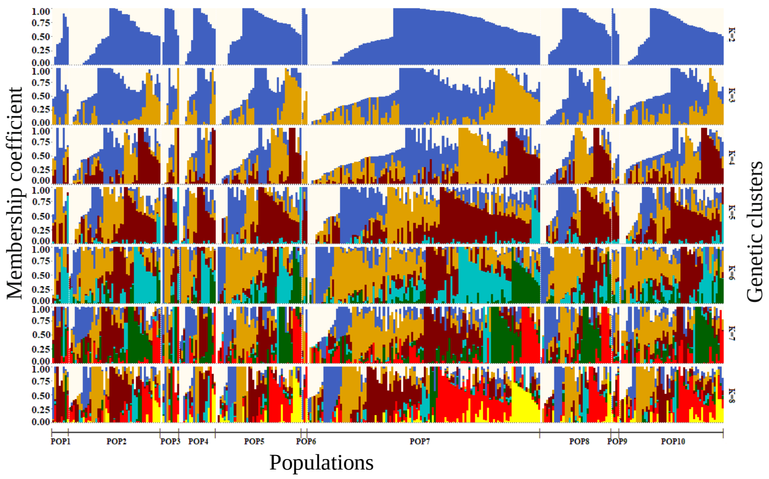

Figure 4. Population genetic structure among 313 Ethiopian sorghum ~~germplasm collections~~genotypes. ~~using the Diversity Array Technology Sequencing based single nucleotide polymorphisms marker analysis.~~ Each entry is represented as a single vertical line partitioned into segments corresponding to the inferred membership according to different K genetic clusters as indicated by the colors. Note: Pop1 to Pop10 represent ~~populations~~ collection area 1 to 10 resolved through GWAS.

A scree plot was generated to visualize the number of principal components. Overall 10 principal components were identified of which principal components 1 (PC1), PC2 and PC3 explained relatively the highest proportion to the total variance (Fig 5A). Principal coordinate analysis (PCA) using PC1 and PC2 stratified the test populations based on areas of collection (Fig 5B). The allocation of test genotypes were irrespective to origin of collections.

(A) (B)

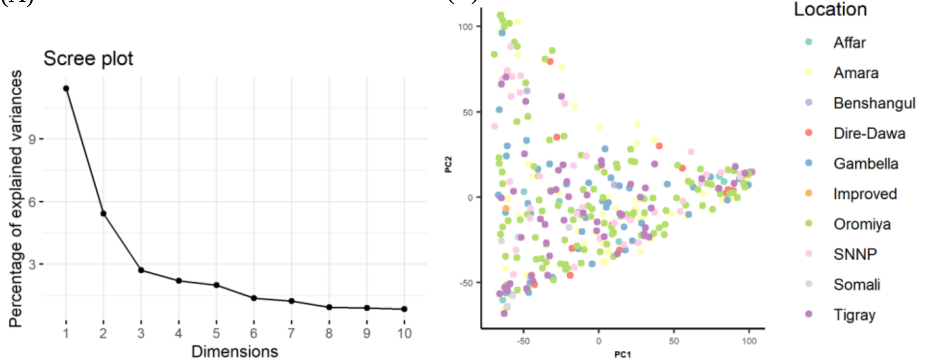

Figure 5. Principal component analysis among 313 sorghum collections based on 11,611 SNPs using the first two principal components. The large proportions of the variances contained in the data are retained by the first three principal components (A), while the relationship among collections between areas of origin is represented in (B).

Marker-trait association

A summary of the genome-wide association analysis of anthracnose resistance and SNP markers amongst 313 sorghum collections is presented in Table 3. A total of eight significant ($P < 0.001$) marker-trait associations were resolved on chromosomes 1, 4, 6, 8, 9 and 10 (Table 3 and Fig 6A). The quantile-quantile plots (Fig 6B) of p-values were examined to determine how well the models accounted for population structure and familial relatedness.

Three markers including markers *rs1887698* and *rs2681689* located on chromosome 9 and *rs1884746* on chromosome 10 had significant ($P < 0.001$) association with anthracnose resistance. Markers *rs1887698* and *rs2681689* significantly associated with chromosome 9 with $P = 4.4 \times 10^{-06}$ and 4.99×10^{-06}, respectively explaining 15.9% of the total variation for anthracnose resistance. Whereas marker *rs1884746* located on chromosome 10 ($P = 4.28 \times 10^{-05}$) explained 15.0% of the total variation. Furthermore, SNPs significantly associated with anthracnose resistance were

detected on chromosomes 1, 8, 9, 6 and 4 which explained 4.86 to 5.23% of the total variation among the tested sorghum collections for anthracnose resistance. Overall, eight SNPs were found affecting anthracnose resistance (Table 3) of which four SNPs were above the cutoff point (Fig 6A). Hence, the significant MTAs localized on the specific chromosomes influence anthracnose resistance and could be exploited in gene introgression and selection. Also, the proportion of the phenotypic variation ($R^2 > 4$) observed for all significant markers suggest their possible influence on anthracnose resistance.

Table 3. Summary on genome-wide association analysis of anthracnose resistance and SNP markers amongst 313 sorghum collections indicating significant markers, alleles detected with chromosome number and position, significant value, coefficient of determination (R^2) and annotation.

Significant marker	Allele	Chromosome number	Position	P- value	R^2	Annotation
rs1887698	A/G	9	4.8E+07	4.40E-06	15.9	Sobic.009G126300 (Threonine-specific protein kinase)
rs2681689	C/T	9	815114	4.99E-06	15.9	Sobic.009G008800 (xylem cysteine proteases)
rs100028710	A/C	4	5.3E+07	1.59E-05	5.23	-
rs1938969	C/T	6	1561991	0.00068	5.21	-
rs1884746	A/G	10	1055302	4.28E-05	15.0	Sobic.010G012200 (Gluconokinase / Gluconate kinase)
rs5196058	C/T	9	4.8E+07	0.00074	4.95	-
rs2205151	T/G	8	5.4E+07	0.00032	4.87	-
rs100052771	A/G	1	2.2E+07	0.00032	4.86	-

- denote not available

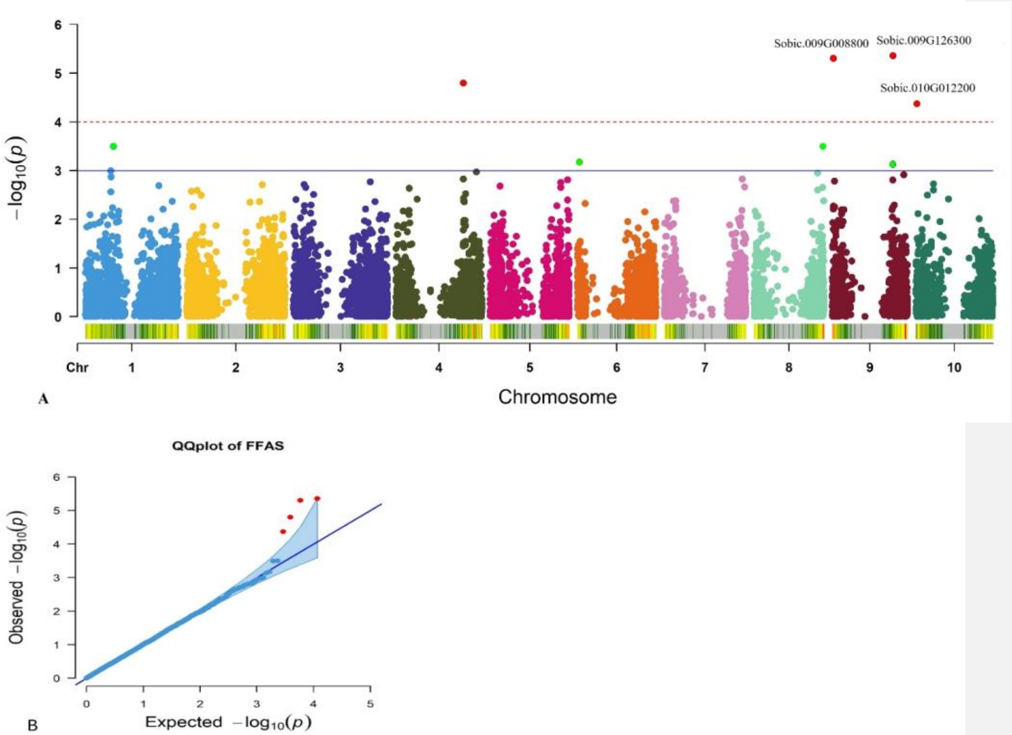

Figure 6. Genome-wide association of anthracnose resistance amongst 313 sorghum collections with 11,643 SNP markers using a FarmCPU Model: (a) Manhattan plots showing significant false discovery rate (FDR) adjusted P-value of ≤0.1 associated with anthracnose resistance and (b) Log Q-Q plots validating the FarmCPU Model and depicting consistency in reducing -log10(p-values) toward the expected level. A dash line represents the threshold from the FDR, and a blue line represents the significant threshold −Log10 (P) value.

Putative genes that condition anthracnose resistance

A blast search of potential anthracnose resistance genes identified three candidate genes. The two candidate genes were located on chromosome 9, while one gene on chromosome 10. The two genes on chromosome 9 are annotated with protein coding genes including *Sobic.009G008800* (*xylem cysteine proteases*) and *Sobic.009G126300 (Threonine-specific protein kinase)* linked to markers rs2681689 and rs1887698, respectively. In addition, one gene was annotated with protein

coding gene *Sobic.010G012200 (Gluconokinase/Gluconate kinase)* and linked to marker rs1884746 located on chromosome 10. The SNP data set used in study is presented in Supplementary Table 2.

Discussion

The existence of significant genotype by x season interaction effects on anthracnose resistance suggests that there was considerable genetic diversity among the assessed genotypes and genotype performance varies across seasons. These results support earlier studies that there is potentially adequate genetic variation among Ethiopian sorghum to select for anthracnose resistance and to support genetic analysis, gene discovery and breeding efforts [8, 9, 11, 38].

Investigating the genetic bases of anthracnose resistance presents an opportunity for resistance breeding. To complement the ongoing need for anthracnose resistant sorghum genetic resources the current study explored the population structure and association of genomic regions with anthracnose resistance in a diverse population of sorghum collections from Ethiopia, its center of origin and diversity. Sorghum anthracnose resistance was confirmed through rigorous field screening in three seasons. Previous studies assessed the genetic basis of anthracnose resistance in sorghum [7]. The authors reported three loci on chromosome 5 amongst 335 US accessions using GWAS. In addition, [10] used 359 sorghum populations and identified quantitative trait loci on chromomere chromosome 8 conditioning resistance to *Colletotrichum sublineola*anthracnose.

Formatted: Font: Not Italic

The population structure generated using hierarchical clustering, admixture, and principal component analysis identified a clear differentiation of the assessed sorghum collections (Figs 4, 5). The test population was grouped into ~~10~~ eight distinct genetic groups (Fig 4). ~~Previous~~ Previous studies of population structure ~~conducted by [12]~~ identified 11 genetic groups using 1,425 Ethiopian sorghum ~~landrace~~ accessions [12]. ~~Furthermore~~In addition, ~~[11] reported 5 populations after evaluating~~ a total of 318 ~~accessions in~~ Sudanese sorghum core ~~sorghum~~ collections were evaluated with 183,144 SNP markers using the model-based clustering method that portrayed five subpopulations [11]. Furthermore, 940 diverse sorghum landraces of Ethiopia were assessed using 54,080 SNP markers that identified 12 subpopulations [48].

Markers associated with anthracnose resistance were identified on chromosomes 9 and 10 suggesting the value of these genomic regions in gene introgression and pyramiding programs. Previously, markers for anthracnose resistance in sorghum collections from US were identified on chromosome 5 [7, 9] and chromosome 9 [8] using SNP markers. The new markers are potentially useful for marker assisted breeding of anthracnose resistance in the assessed sorghum populations [39].

The current study identified eight significant (P < 0.001) marker-trait associations on chromosomes 1, 4, 6, 8, 9 and 10 (Table 3 and Fig 6A). The quantile-quantile plots (Fig 6B) showed a perfect fit of the observed and expected population structures for anthracnose resistance using the FarmCPU Model. The novel genes are additions to previously identified genomic regions conditioning resistance to anthracnose, downy mildew, and head smut in sorghum [4,7-9]. Four SNP markers above the cutoff point (Fig 6A) are novel anthracnose resistance markers detected in

Formatted: Font: Not Italic

Formatted: Font: Not Italic

Formatted: Font: Not Italic

Formatted: Font: Not Italic

this study. The markers are located on chromosomes 9, 10 and 4. Ahn et al. ~~Previously~~ [4] used 242 mini core accessions and three sorghum cultivars and subjected to genome wide association analysis. The authors reported anthracnose resistance gene on chromosome 8. ~~Also~~ In addition, Cruet-Burgos et al. [9] examined 114 recombinant inbred lines (RILs) obtained from crosses of sorghum anthracnose resistant line SC112-14 (PI533918) and susceptible line PI609251 and identified tightly linked anthracnose resistance locus on chromosome 5.

Through blast analysis the present study identified three candidate anthracnose resistance genes. The two genes are located on chromosome 9, while one gene on chromosome 10. The two genes located on chromosome 9 are annotated with protein coding genes *Sobic.009G008800* and *Sobic.009G126300* linked to markers rs1887698 and rs2681689, respectively. The *Sobic.009G008800* reportedly had strong association with annotated function xylem cysteine peptidase 1 of sorghum conditioning disease resistance. The biological effect of the gene includes programmed cell death, responsive to dehydration, while the annotated sorghum genes had roles in anthracnose resistance on chromosome 9 [40, 41]. The authors reported that cysteine proteases is one of the first Arabidopsis proteases functioning in the immune system of sorghum. In a mutant strain of Arabidopsis, over expression of the aspartic protease Constitutive Disease Resistance 1 (CDR1) resulted in resistance to the virulent strain of *Pseudomonas syringae*, a bacterium that causes diseases of monocots, herbaceous dicots, and woody dicots. Cysteine proteases also contain a cysteine nucleophilic residue that performs a nucleophilic attack proteolysis resulting in an intermediate state where the enzyme is covalently attached to its substrate [42]. Cysteine proteases reportedly conferred immunity in tomato against diseases caused by *Phytophthora infestans* (Pinf) and *Cladosporium fulvum* (syn. Passalora fulva) [43, 44].

In the present study a second significant candidate gene, *Sobic.009G126300* was located on chromosome 9 linked to marker rs1887698 with annotated function similar to Serine/threonine-protein kinase CTR1. Serine/threonine-protein kinase is reportedly known for mediating drought stress tolerance in plants. Furthermore, threonine-protein kinase is believed to have a role in regulating ethylene hormone pathways conditioning drought response in senna (*Cassia angustifolia* Vahl.) [45]. The 3rd candidate gene identified in this study linked to the marker rs1884746 annotated with *Sobic.010G012200 (Gluconokinase/Gluconate kinase).* Gluconate kinase is an essential enzyme of the oxidative pentose phosphate pathway which is responsible in NADPH supplies during fatty acid synthesis in developing embryos of *Thermotoga maritima* [46, 47].

The anthracnose resistance genes identified after validation will provide ideal molecular markers to speed resistance breeding. The genes would be directly used marker assisted selection through screening for the presence of combinations of QTLs could lead to a more durable form of resistance.

Formatted: Font: Not Bold

Formatted: Font: Not Bold

Formatted: Justified

Conclusion

Marker trait association is key to identify genomic regions that are associated with anthracnose resistance for marker-assisted breeding in sorghum improvement programs. The present study identified four novel markers associated with anthracnose resistance designated as rs1887698, rs2681689, rs1884746 and rs100028710. The new genetic markers identified in the current sorghum populations are useful genomic resources for future parental selection, quantitative trait loci analysis, trait introgression, gene pyramiding and marker-assisted ~~breeding~~ selection of

anthracnose resistance in sorghum breeding programs in Ethiopia or related agro-ecologies. Further studies are required to validate the significant markers identified in the present study.

Acknowledgments

The authors thank the National Research Foundation (NRF)/South Africa for financial support. The Integrated Genotyping Service and Support (IGSS) platform of the Biosciences Eastern and Central Africa (BecA-ILRI)/Kenya is acknowledged for genotyping the sorghum accessions.

Author Contributions

Conceptualization: GM HS DL.

Data curation: GM HS DL.

Formal analysis: GM HS EA DL.

Funding acquisition: GM HS DL.

Investigation: GM HS DL.

Methodology: GM HS EA DL.

Project administration: GM HS DL.

Resources: GM HS EA DL.

Writing - original draft: GM HS EA.

Writing - review & editing: GM HS EA DL.

References

1. Dahlberg J, Berenji J, Sikora V, Latkovic D. Assessing sorghum [*Sorghum bicolor* (L) Moench] germplasm for new traits: food, fuels ~~&~~ and unique uses. Maydica. 2011; 56:85–92.

2. Nida H, Girma G, Mekonen M, Lee S, Seyoum A, Dessalegn K, et al. Identification of sorghum grain mold resistance loci through genome wide association mapping. Journal of Cereal Science. 2019; 85:295–304.

3. Ejeta G, Gressel J. Integrating new technologies for striga control: towards ending the witch-hunt: World Scientific Publishing; 2007. 345pp.

4. Ahn E, Hu Z, Perumal R, Prom LK, Odvody G, Upadhyaya HD, et al. Genome wide association analysis of sorghum mini core lines regarding anthracnose, downy mildew, and head smut. PLOS ONE. 2019; 14:e0216671.

5. Chala A, Brurberg MB, Tronsmo AM. Prevalence and intensity of sorghum anthracnose in Ethiopia. Journal of SAT Agricultural Research. 2007; 5:145–298.

6. Thakur RP, Mathur K. Anthracnose. In: Compendium of sorghum diseases. (Eds. R. A. Frederiksen and G. N. Odvody). The American Phytopathological Society, St Paul, MN, USA; 2000. pp:10–12.

7. Cuevas HE, Prom LK, Cooper EA, Knoll JE, Ni X. Genome- wide association mapping of anthracnose (*Colletotrichum sublineolum*) resistance in the US sorghum association panel. The Plant Genome. 2018; 11:170099. https://doi.org/10.3835/plantgenome2017.11.0099

8. Cuevas HE, Prom LK, Cruet-Burgos CM. Genome-wide association mapping of anthracnose (*Colletotrichum sublineolum*) resistance in NPGS Ethiopian sorghum germplasm. G3: Genes, Genomes, Genetics. 2019; 9:2879–2885. https://doi.org/10.1534/g3.119.400350

9. Cruet-Burgos CM, Cuevas HE, Prom LK, Knoll JE, Stutts LR, Vermerris W. Genomic dissection of anthracnose (*Colletotrichum sublineolum*) resistance response in sorghum differential line SC112-14. G3: Genes, Genomes, Genetics. 2020; 10:1403–1412. https://doi.org/10.1534/g3.120.401121

10. Prom LK, Ahn E, Isakeit T, Magill C. GWAS analysis of sorghum association panel lines identifies SNPs associated with disease response to Texas isolates of *Colletotrichum sublineola*. Theoretical and Applied Genetics. 2019; 132:1389–1396. https://doi.org/10.1007/s00122-019-03285

11. Cuevas HE, Prom LK. Evaluation of genetic diversity, agronomic traits, and anthracnose resistance in the NPGS Sudan sorghum core collection. BMC Genomics. 2020; 21:88. https://doi.org/10.1186/s12864-020-6489-0

12. Girma G, Nida H, Seyoum A, Mekonen M, Nega A, Lule D, et al. A large-scale genome-wide association analyses of Ethiopian sorghum landrace collection reveal loci associated with important traits. Frontiers in Plant Science. 2019; 10:691. https://doi.org/10.3389/fpls.2019.00691

13. Rhodes DH, Hoffmann Jr L, Rooney WL, Ramu P, Morris GP, Kresovich S. Genome-wide association study of grain polyphenol concentrations in global sorghum [*Sorghum bicolor* (L.) Moench] germplasm. Journal of Agricultural and Food Chemistry. 2014; 62: 10916–10927. https://doi.org/10.1021/jf503651t

14. Batley J, Edwards D. SNP applications in plants. Association mapping in plants: Springer; 2007. pp:95-102.

15. Chen J, Chopra R, Hayes C, Morris G, Marla S, Burke J, et al. Genome- wide association study of developing leaves' heat tolerance during vegetative growth stages in a sorghum association panel. The Plant Genome. 2017; 10(2):1–15. https://doi.org/10.3835/plantgenome2016.09.0091

16. Lipka AE, Tian F, Wang Q, Peiffer J, Li M, Bradbury PJ, et al. GAPIT: Genome association and prediction integrated tool. Bioinformatics. 2012; 28(18):2397-2399. https://doi:10.1093/bioinformatics/bts444

17. Vavilov NI. ~~(1951)~~ The origin, variation, immunity and breeding of cultivated plants. Translated by Chester K.S. Ronald Press, New York; 1951. 366pp.

18. Doggett H. Sorghum. Longman Scientific ~~&~~ and Technical, London; 1988.

19. Ayana A, Bekele E. Geographical patterns of morphological variation in sorghum (*Sorghum bicolor* (L.) Moench) germplasm from Ethiopia and Eritrea: quantitative characters. Euphytica. 2000; 115:91–104.

20. Smith CW, Frederiksen RA. Sorghum: origin, history, technology, and production: John Wiley & and Sons, USA; 2000. pp.840.

21. Paterson AH, Bowers JE, Bruggmann R, Dubchak I, Grimwood J, Gundlach H, et al. The *Sorghum bicolor* genome and the diversification of grasses. Nature. 2009; 457:551–556.

22. Patil NY, Klein RR, Williams CL, Collins SD, Knoll JE, Burrell AM, et al. Quantitative trait loci associated with anthracnose resistance in sorghum. Crop Science. 2017; 57: 877–890.

23. Rhodes DH, Hoffmann L, Rooney WL, Herald TJ, Bean S, Boyles R, et al. Genetic architecture of kernel composition in global sorghum germplasm. BMC Genomics. 2017; 18:1–8. https://doi.org/10.1186/s12864-016-3403-x

24. Prom LK, Perumal R, Erpelding JE, Isakeit T, Montes-Garcia N, Magill CW. A pictorial technique for mass screening of sorghum germplasm for anthracnose (*Colletotrichum sublineolum*) resistance. The Open Agriculture Journal. 2009; 3:20–25.

25. Chala A, Tronsmo AM. Evaluation of Ethiopian sorghum accessions for resistance against *Colletotrichum sublineolum*. European Journal of Plant Pathology. 2012; 132:179–189.

26. Campbell CL, Madden LV. Introduction to Plant Disease Epidemiology: John Wiley & and Sons; 1990. pp.560.

27. Matschegewski C, Zetzsche H, Hasan Y, Leibeguth L, Briggs W, Ordon F. Genetic variation of temperature-regulated curd induction in cauliflower: elucidation of floral transition by genome-wide association mapping and gene expression analysis. Frontiers in Plant Science. 2015; 6:1–14. https://doi.org/10.3389/fpls.2015.00720

28. SAS. Statistical analysis system, version 9.0. Cary, NC: SAS Institute Inc; 2012.

29. DArT. Extraction protocol for DArT. Camberra, Australia: Diversity Arrays Technology; 2014.

30. Wickham H. Elegant graphics for data analysis (ggplot2). Applied Spatial Data Analysis R; 2009.

31. Alexander DH, Lange K. Enhancements to the ADMIXTURE algorithm for individual ancestry estimation. BMC Bioinformatics. 2011; 12:1–6. https://doi.org/10.1186/1471-2105-12-246

32. Francis RM. Pophelper: an R package and web app to analyse and visualize population structure. Molecular Ecology Resources. 2017; 17:27–32. https://doi.org/10.1111/1755-0998.12509

33. Liu X, Huang M, Fan B, Buckler ES, Zhang Z. Iterative usage of fixed and random effect models for powerful and efficient genome-wide association studies. PLOS Genetics. 2016; 12:e1005767. https://doi.org/10.1371/journal.pgen.1005767

34. VanRaden PM. Efficient methods to compute genomic predictions. Journal of Dairy Science. 2008; 91:4414–4423.

35. Zhao X, Han Y, Li Y, Liu D, Sun M, Zhao Y, et al. Loci and candidate gene identification for resistance to *Sclerotinia sclerotiorum* in soybean (*Glycine max* L. Merr.) via association and linkage maps. The Plant Journal. 2015; 82:245–255. https://doi.org/10.1111/tpj.12810

36. Benjamini Y, Hochberg Y. Controlling the false discovery rate: a practical and powerful approach to multiple testing. Journal of the Royal Statistical Society: series B (Methodological). 1995; 57:289–300.

37. McCormick RF, Truong SK, Sreedasyam A, Jenkins J, Shu S, Sims D, et al. The sorghum bicolor reference genome: improved assembly, gene annotations, a transcriptome atlas, and signatures of genome organization. The Plant Journal. 2018; 93:338–354.

38. ~~Hugo E.~~ Cuevas HE, Louis K. Prom, Elizabeth A. Cooper, Joseph E. Knoll a, Ni X. Genome-wide association mapping of anthracnose (*Colletotrichum sublineolum*) resistance in the U.S. sorghum association panel. The Plant Genome. 2018; 11:13.

39. Kamfwa K, Cichy KA, Kelly JD. Genome-wide association study of agronomic traits in common bean. The Plant Genome. 2015; 8(2):1–12.

40. Pogány M, Dankó T, Kámán-Tóth E, Schwarczinger I, Bozsó Z. Regulatory proteolysis in Arabidopsis-pathogen interactions. International Journal of Molecular Sciences. 2015; 16: 23177–23194. https://doi.org/10.3390/ijms161023177 PMID: 26404238

41. Patil NY. Investigating the genetic basis of agronomically important traits in sorghum. A Dissertation; 2016. pp.140.

42. Van der Hoorn RA, Leeuwenburgh MA, Bogyo M, Joosten MH, Peck SC. Activity profiling of papain-like cysteine proteases in plants. Plant Physiology. 2004; 135:1170–1178. https://doi.org/10.1104/pp.104.041467

43. Shindo T, Misas-Villamil JC, Hörger AC, Song J, van der Hoorn RA. A role in immunity for Arabidopsis cysteine protease RD21, the ortholog of the tomato immune protease C14. PLOS ONE. 2012; 7:e29317. https://doi.org/10.1371/journal.pone.0029317

44. van Esse HP, Van't Klooster JW, Bolton MD, Yadeta KA, Van Baarlen P, Boeren S, et al. The *Cladosporium fulvum* virulence protein Avr2 inhibits host proteases required for basal defense. The Plant Cell. 2008; 20:1948–1963. https://doi.org/10.1105/tpc.108.059394

45. Mehta RH, Ponnuchamy M, Kumar J, Reddy NRR. Exploring drought stress-regulated genes in senna (*Cassia angustifolia* Vahl.): a transcriptomic approach. Functional and Integrative Genomics. 2017; 17:1–25. https://doi.org/10.1007/s10142-016-0523-y

46. Zhang Y, Zagnitko O, Rodionova I, Osterman A, Godzik A. The FGGY carbohydrate kinase family: insights into the evolution of functional specificities. PLOS Computational Biology. 2011; 7:e1002318. https://doi.org/10.1371/journal.pcbi.1002318

47. Dos Santos Fernandes L, Correa FM, Ingram KT, de Almeida A-AF, Royaert S. QTL mapping and identification of SNP-haplotypes affecting yield components of *Theobroma cacao* L. Horticulture Research. 2020; 7:1–18.

48. Menamo, T, Kassahun B, Borrell A, Jordan D, Tao Y, Hunt C, Mace E. Genetic diversity of Ethiopian sorghum reveals signatures of climatic adaptation. Theoretical and Applied Genetics. 2021; 134: 731–742.

Formatted: Font: Not Bold

Formatted: Font: Not Bold

27 June 2021

Dr. Karthikeyan Adhimoolam,

Academic Editor, PLOS ONE

Dear Dr. Adhimoolam ,

Rebuttal letter in response to the comments forwarded by the reviewers on manuscript number, PONE-D-21-10117 [Genome-wide association analysis of anthracnose resistance in sorghum [*Sorghum bicolor* (L.) Moench].

We are grateful for the constructive feedback and invaluable inputs given by reviewers. These have assisted in improving the quality of our manuscript.

We have promptly integrated the required changes in the present submission, all according to the comments given by the reviewers. The changes made in the manuscript are shown in track change mode. Below please find a point-by-point response to each comment given by the reviewers.

Thank you in advance for considering our contribution in PLOS ONE.

Sincerely,

Girma Mengistu, PhD

Corresponding author

Manuscript number, PONE-D-21-10117

Reviewers' comments:

Reviewer's Responses to Questions

Comments to the Author

1. Is the manuscript technically sound, and do the data support the conclusions?

The manuscript must describe a technically sound piece of scientific research with data that supports the conclusions. Experiments must have been conducted rigorously, with appropriate controls, replication, and sample sizes. The conclusions must be drawn appropriately based on the data presented.

Reviewer #1: Partly

Reviewer #2: Yes

Response 1. Thank you for the constructive feedback. The manuscript is based on integrated data sets through field-based phenomic and genomic analyses which supported the final conclusions and recommendations. The field experiments were conducted through rigorous data location across three consecutive seasons involving sufficiently large number of sorghum germplasm collections and anthracnose susceptible and resistant standard controls. The conclusions drawn from the study were in synchronization with the phenomic and genomic data and sound genetic analyses.

---

2. Has the statistical analysis been performed appropriately and rigorously?

Reviewer #1: Yes

Reviewer #2: Yes

Response 2. Thank you for the comment. The statistical analysis has been performed appropriately and rigorously.

---

3. Have the authors made all data underlying the findings in their manuscript fully available?

The PLOS Data policy requires authors to make all data underlying the findings described

in their manuscript fully available without restriction, with rare exception (please refer to the Data Availability Statement in the manuscript PDF file). The data should be provided as part of the manuscript or its supporting information, or deposited to a public repository. For example, in addition to summary statistics, the data points behind means, medians and variance measures should be available. If there are restrictions on publicly sharing data—e.g. participant privacy or use of data from a third party—those must be specified.

Reviewer #1: No

Reviewer #2: Yes

Response 3. The comment is accepted. Both data sets (genotypic and anthracnose severity data) are provided as a Supplementary Table 1 and 2.

---

4. Is the manuscript presented in an intelligible fashion and written in standard English?

PLOS ONE does not copyedit accepted manuscripts, so the language in submitted articles must be clear, correct, and unambiguous. Any typographical or grammatical errors should be corrected at revision, so please note any specific errors here.

Reviewer #1: No

Reviewer #2: Yes

Response 4. Thank you for the comments. The authors have checked the manuscript and typographical or grammatical errors are promptly fixed.

---

5. Review Comments to the Author

Please use the space provided to explain your answers to the questions above. You may also include additional comments for the author, including concerns about dual publication, research ethics, or publication ethics. (Please upload your review as an attachment if it exceeds 20,000 characters)

Reviewer #1: Genome wide association analysis of anthracnose resistance in sorghum [*Sorghum bicolor* (L.) Moench]

This manuscript includes the anthracnose resistance evaluation of 366 Ethiopian accessions over three years and the genotyping characterization of these accessions

based on DArT sequencing markers. Later the authors analyzed the population structure of this Ethiopian accessions and performed GWAS to identify anthracnose resistance loci.

Definitely, this manuscript has valuable information for sorghum breeding program in Ethiopia and other countries. However, the authors need to improve the manuscript to be accepted for publications. At this stage, the manuscript has many concerns that need to be addressed, and I suggest to the authors to resubmit the manuscript after its improvement.

1- The English of the manuscript need to be improved. It has multiple typo errors and sentences that need to be rewrite for clarification. The use of number in reference needs to be verified in the sentences because many are difficult to read. Eg. Also [9] examined 114 recombinant inbred lines……

Response 1. As advised, the use of number(s) in the reference have been revised. This will ease to reading the references in the manuscript.

2- The evaluation of anthracnose is not well explained in the manuscript. They just mention is acording to Chala et al. 2012. They need to provide some information of how they did the scoring.

Response 2. The comment is accepted. We have added one paragraph.

3- Candidate genes were identified based in the SNPs position. They authors should explain the window size (20Kb upstream/downstream of the SNP) used to identity candidate genes.

Response 3. Thank you for the insight. This was achieved using the physical genome assembly of sorghum reference genome sequence, version 3.1.1 (https://phytozome.jgi.doe.gov/pz/portal.html#!info?alias=Org *Sbicolor*) serving as identifying candidate genes between 20 kb on either side of significant SNPs.

4- They authors should provide a supplementary table with the anthracnose score of each accession. Also make available the DArT genotyping.

Response 4. Data on anthracnose score for each accession and DArT genotyping are provided as Supplementary Table 1 and 2.

5- The authors never mentioned how many accessions were finally classified as resistant to anthracnose. I am wondering if they did a means compare analysis, contrast, or any other statistical analysis to classify an accession resistant to anthracnose.

Response 5. Thank you for the comment. 63 accessions were identified as moderately resistant to anthracnose disease. This is indicated in the Results section.

6- Population structure analysis is confuse. They first mention the optimal number is 8, then in the second paragraph they mention 10.

Response 6. The error is corrected, and the number of sub-populations are 8.

7- The marker trait association is confuse. In Mat and Met they indicate they will use the FDR p values less than 0.1 according to Benjamini and Hochberg. Then in results they mentioned eight significant SNPs and highlight that 4 were above the cutoff point. Do you have two threshold? You mentioned just one in Mat and Met.

Response 7. Thank you. We have added a description in Fig 4. A dash line represents the threshold from the FDR, and a blue line represents the significant threshold −Log10 (P) value.

8- The discussion is poor. Previous studies based on Ethiopian germplasm identified an anthracnose resistant locus in chromosome 9. How distance is the DArT markers from this locus?

Response 8. The distance from previously identified Ethiopian germplasm an anthracnose resistant locus in chromosome 9 is 360Kb.

9- The discussion don't include the population structure analysis of other Ethiopian germplasm. Eg. Cuevas et al. 2017 BMC Genomics, Girma et al. 2019, Menamo et al. 2021 TAG,

Response 9. Thank you for this input. We have revised as follows: Previous studies of population structure identified 11 genetic groups using 1,425 Ethiopian sorghum accessions [12]. In addition, a total of 318 Sudanese sorghum core collections were evaluated with 183,144 SNP markers using the model-based clustering method that portrayed five subpopulations [11]. Furthermore, 940 diverse sorghum landraces of Ethiopia were assessed using 54,080 SNP markers that identified 12 subpopulations [48].

10 – Most of the discussion is centralized in candidate genes, instead of how to use the information for breeding and the conservation of sorghum germplasm.

Response 10. The anthracnose resistance genes identified in the present study will be validated across multiple seasons and locations to serves as ideal molecular markers for anthracnose resistance breeding programs. The novel genes could be pyramided into anthracnose susceptible sorghum lines through marker-assisted selection. A combinations of QTLs could render durable resistance to sorghum anthracnose.

11- Reference num 38 have an incorrect format. Last name must be first.

Response 11. Accepted. We have corrected the reference number 38 in the manuscript.

Reviewer #2: The manuscript entitled "Genome-wide association analysis of anthracnose resistance in sorghum [Sorghum bicolor (L.) Moench]" was written well and identified four novel markers (rs1887698, rs2681689, rs1884746 and rs100028710) associated with anthracnose resistance. Also by using the reference genome, authors identified the putative functional candidate genes annotated with genes in other species such as Arabidopsis and tomato. Population structure analysis with the clustering of lines and GWAS study for marker-trait association is well documented. However, the manuscript will be completed only if the identified markers are validated with additional study. To accelerate the breeding process for anthracnose resistance, only validated markers are more useful for large scale screening. Hence, authors are requested to provide the additional information on the identified markers' validation.

Response. Thank you for the encouraging comment. Our next plan is to validate the identified four novel markers and implement marker-assisted breeding to stack the genes in a desirable genetic background.

---

6. PLOS authors have the option to publish the peer review history of their article (what does this mean?). If published, this will include your full peer review and any attached files.

Response 6. Yes

If you choose "no", your identity will remain anonymous but your review may still be made public.

Do you want your identity to be public for this peer review? For information about this choice, including consent withdrawal, please see our Privacy Policy.

Reviewer #1: No

Reviewer #2: No

[NOTE: If reviewer comments were submitted as an attachment file, they will be attached to this email and accessible via the submission site. Please log into your

account, locate the manuscript record, and check for the action link "View Attachments". If this link does not appear, there are no attachment files.]

While revising your submission, please upload your figure files to the Preflight Analysis and Conversion Engine (PACE) digital diagnostic tool, https://pacev2.apexcovantage.com/. PACE helps ensure that figures meet PLOS requirements. To use PACE, you must first register as a user. Registration is free. Then, login and navigate to the UPLOAD tab, where you will find detailed instructions on how to use the tool. If you encounter any issues or have any questions when using PACE, please email PLOS at figures@plos.org. Please note that Supporting Information files do not need this step.
