# Supplementary material for: Genome-wide association analysis of anthracnose resistance in sorghum [Sorghum bicolor (L.) Moench]
Source: PLoS One. 2021 Dec 20;16(12):e0261461. doi: 10.1371/journal.pone.0261461 (PMC8687563; doi:10.1371/journal.pone.0261461)
Supplement: Attachment — Submitted filename: Response to Reviewers PONE-D-21-10117.docx [file pone.0261461.s003.docx]

27 June 2021

Dr. Karthikeyan Adhimoolam,

Academic Editor, PLOS ONE

Dear Dr. Adhimoolam ,

Rebuttal letter in response to the comments forwarded by the reviewers on manuscript number, PONE-D-21-10117 [Genome-wide association analysis of anthracnose resistance in sorghum [*Sorghum bicolor* (L.) Moench].

We are grateful for the constructive feedback and invaluable inputs given by reviewers. These have assisted in improving the quality of our manuscript.

We have promptly integrated the required changes in the present submission, all according to the comments given by the reviewers. The changes made in the manuscript are shown in track change mode. Below please find a point-by-point response to each comment given by the reviewers.

Thank you in advance for considering our contribution in PLOS ONE.

Sincerely,

Girma Mengistu, PhD

Corresponding author

Manuscript number, PONE-D-21-10117

Reviewers' comments:

Reviewer's Responses to Questions

**Comments to the Author**

1. Is the manuscript technically sound, and do the data support the conclusions?

The manuscript must describe a technically sound piece of scientific research with data that supports the conclusions. Experiments must have been conducted rigorously, with appropriate controls, replication, and sample sizes. The conclusions must be drawn appropriately based on the data presented.

Reviewer #1: Partly

Reviewer #2: Yes

**Response 1.** Thank you for the constructive feedback. The manuscript is based on integrated data sets through field-based phenomic and genomic analyses which supported the final conclusions and recommendations. The field experiments were conducted through rigorous data location across three consecutive seasons involving sufficiently large number of sorghum germplasm collections and anthracnose susceptible and resistant standard controls. The conclusions drawn from the study were in synchronization with the phenomic and genomic data and sound genetic analyses.

2. Has the statistical analysis been performed appropriately and rigorously?

Reviewer #1: Yes

Reviewer #2: Yes

Response 2. Thank you for the comment. The statistical analysis has been performed appropriately and rigorously.

3. Have the authors made all data underlying the findings in their manuscript fully available?

The [PLOS Data policy](http://www.plosone.org/static/policies.action#sharing) requires authors to make all data underlying the findings described in their manuscript fully available without restriction, with rare exception (please refer to the Data Availability Statement in the manuscript PDF file). The data should be provided as part of the manuscript or its supporting information, or deposited to a public repository. For example, in addition to summary statistics, the data points behind means, medians and variance measures should be available. If there are restrictions on publicly sharing data—e.g. participant privacy or use of data from a third party—those must be specified.

Reviewer #1: No

Reviewer #2: Yes

Response 3. The comment is accepted. Both data sets (genotypic and anthracnose severity data) are provided as a Supplementary Table 1 and 2.

 4. Is the manuscript presented in an intelligible fashion and written in standard English?

PLOS ONE does not copyedit accepted manuscripts, so the language in submitted articles must be clear, correct, and unambiguous. Any typographical or grammatical errors should be corrected at revision, so please note any specific errors here.

 Reviewer #1: No

Reviewer #2: Yes

Response 4. Thank you for the comments. The authors have checked the manuscript and typographical or grammatical errors are promptly fixed.

5. Review Comments to the Author

Please use the space provided to explain your answers to the questions above. You may also include additional comments for the author, including concerns about dual publication, research ethics, or publication ethics. (Please upload your review as an attachment if it exceeds 20,000 characters)

Reviewer #1: Genome wide association analysis of anthracnose resistance in sorghum [*Sorghum bicolor* (L.) Moench]

This manuscript includes the anthracnose resistance evaluation of 366 Ethiopian accessions over three years and the genotyping characterization of these accessions based on DArT sequencing markers. Later the authors analyzed the population structure of this Ethiopian accessions and performed GWAS to identify anthracnose resistance loci.

Definitely, this manuscript has valuable information for sorghum breeding program in Ethiopia and other countries. However, the authors need to improve the manuscript to be accepted for publications. At this stage, the manuscript has many concerns that need to be addressed, and I suggest to the authors to resubmit the manuscript after its improvement.

1- The English of the manuscript need to be improved. It has multiple typo errors and sentences that need to be rewrite for clarification. The use of number in reference needs to be verified in the sentences because many are difficult to read. Eg. Also [9] examined 114 recombinant inbred lines……

**Response 1**. As advised, the use of number(s) in the reference have been revised. This will ease to reading the references in the manuscript.

2- The evaluation of anthracnose is not well explained in the manuscript. They just mention is acording to Chala et al. 2012. They need to provide some information of how they did the scoring.

**Response 2**. The comment is accepted. We have added one paragraph.

3- Candidate genes were identified based in the SNPs position. They authors should explain the window size (20Kb upstream/downstream of the SNP) used to identity candidate genes.

**Response 3**. Thank you for the insight. This was achieved using the physical genome assembly of sorghum reference genome sequence, version 3.1.1 (https://phytozome.jgi.doe.gov/pz/portal.html#!info?alias=Org *Sbicolor*) serving as identifying candidate genes between 20 kb on either side of significant SNPs.

4- They authors should provide a supplementary table with the anthracnose score of each accession. Also make available the DArT genotyping.

**Response 4**. Data on anthracnose score for each accession and DArT genotyping are provided as Supplementary Table 1 and 2.

5- The authors never mentioned how many accessions were finally classified as resistant to anthracnose. I am wondering if they did a means compare analysis, contrast, or any other statistical analysis to classify an accession resistant to anthracnose.

**Response 5**. Thank you for the comment. 63 accessions were identified as moderately resistant to anthracnose disease. This is indicated in the Results section.

6- Population structure analysis is confuse. They first mention the optimal number is 8, then in the second paragraph they mention 10.

**Response 6**. The error is corrected, and the number of sub-populations are 8.

7- The marker trait association is confuse. In Mat and Met they indicate they will use the FDR p values less than 0.1 according to Benjamini and Hochberg. Then in results they mentioned eight significant SNPs and highlight that 4 were above the cutoff point. Do you have two threshold? You mentioned just one in Mat and Met.

**Response 7**. Thank you. We have added a description in Fig 4. A dash line represents the threshold from the FDR, and a blue line represents the significant threshold −Log10 (P) value.

8- The discussion is poor. Previous studies based on Ethiopian germplasm identified an anthracnose resistant locus in chromosome 9. How distance is the DArT markers from this locus?

**Response 8**. The distance from previously identified Ethiopian germplasm an anthracnose resistant locus in chromosome 9 is 360Kb.

9- The discussion don’t include the population structure analysis of other Ethiopian germplasm. Eg. Cuevas et al. 2017 BMC Genomics, Girma et al. 2019, Menamo et al. 2021 TAG,

**Response 9**. Thank you for this input. We have revised as follows: Previous studies of population structure identified 11 genetic groups using 1,425 Ethiopian sorghum accessions [12]. In addition, a total of 318 Sudanese sorghum core collections were evaluated with 183,144 SNP markers using the model-based clustering method that portrayed five subpopulations [11]. Furthermore, 940 diverse sorghum landraces of Ethiopia were assessed using 54,080 SNP markers that identified 12 subpopulations [48].

10 – Most of the discussion is centralized in candidate genes, instead of how to use the information for breeding and the conservation of sorghum germplasm.

**Response 10**. The anthracnose resistance genes identified in the present study will be validated across multiple seasons and locations to serves as ideal molecular markers for anthracnose resistance breeding programs. The novel genes could be pyramided into anthracnose susceptible sorghum lines through marker-assisted selection. A combinations of QTLs could render durable resistance to sorghum anthracnose.

11- Reference num 38 have an incorrect format. Last name must be first.

**Response 11**. Accepted. We have corrected the reference number 38 in the manuscript.

Reviewer #2: The manuscript entitled “Genome-wide association analysis of anthracnose resistance in sorghum [Sorghum bicolor (L.) Moench]” was written well and identified four novel markers (rs1887698, rs2681689, rs1884746 and rs100028710) associated with anthracnose resistance. Also by using the reference genome, authors identified the putative functional candidate genes annotated with genes in other species such as Arabidopsis and tomato. Population structure analysis with the clustering of lines and GWAS study for marker-trait association is well documented. However, the manuscript will be completed only if the identified markers are validated with additional study. To accelerate the breeding process for anthracnose resistance, only validated markers are more useful for large scale screening. Hence, authors are requested to provide the additional information on the identified markers’ validation.

**Response**. Thank you for the encouraging comment. Our next plan is to validate the identified four novel markers and implement marker-assisted breeding to stack the genes in a desirable genetic background.

6. PLOS authors have the option to publish the peer review history of their article ([what does this mean?](https://journals.plos.org/plosone/s/editorial-and-peer-review-process#loc-peer-review-history)). If published, this will include your full peer review and any attached files.

**Response 6**. Yes

If you choose “no”, your identity will remain anonymous but your review may still be made public.


**Do you want your identity to be public for this peer review?** For information about this choice, including consent withdrawal, please see our [Privacy Policy](https://www.plos.org/privacy-policy).

Reviewer #1: No

Reviewer #2: No

[NOTE: If reviewer comments were submitted as an attachment file, they will be attached to this email and accessible via the submission site. Please log into your account, locate the manuscript record, and check for the action link "View Attachments". If this link does not appear, there are no attachment files.]

While revising your submission, please upload your figure files to the Preflight Analysis and Conversion Engine (PACE) digital diagnostic tool, <https://pacev2.apexcovantage.com/>. PACE helps ensure that figures meet PLOS requirements. To use PACE, you must first register as a user. Registration is free. Then, login and navigate to the UPLOAD tab, where you will find detailed instructions on how to use the tool. If you encounter any issues or have any questions when using PACE, please email PLOS at [figures@plos.org](mailto:figures@plos.org). Please note that Supporting Information files do not need this step.
