# Supplementary material for: Genome-wide association analysis of anthracnose resistance in sorghum [Sorghum bicolor (L.) Moench]
Source: PLoS One. 2021 Dec 20;16(12):e0261461. doi: 10.1371/journal.pone.0261461 (PMC8687563; doi:10.1371/journal.pone.0261461)
Supplement: Attachment — Submitted filename: Response to Reviewers PONE-D-21-10117.docx [file pone.0261461.s005.docx]

28 th October 2021

Dr Karthikeyan Adhimoolam,

Academic Editor, PLOS ONE

Dear Dr Adhimoolam,

Rebuttal letter in response to the comments forwarded by the reviewers on the manuscript number, PONE-D-21-10117 [Genome-wide association analysis of anthracnose resistance in sorghum [*Sorghum bicolor* (L.) Moench].

We are grateful for the constructive feedback and invaluable inputs given by reviewers. These have assisted in improving the quality of our manuscript.

We have promptly integrated the required changes in the present submission, all according to the comments given by the reviewers. The changes made in the manuscript are shown in blue colour. Below please find a point-by-point response to each comment given by the reviewers.

Thank you in advance for considering our contribution to PLOS ONE.

Sincerely,

Girma Mengistu, PhD

Corresponding author

Manuscript number, PONE-D-21-10117

Reviewers' comments:

Reviewer's Responses to Questions

**Comments to the Author**

1. If the authors have adequately addressed your comments raised in a previous round of review and you feel that this manuscript is now acceptable for publication, you may indicate that here to bypass the “Comments to the Author” section, enter your conflict of interest statement in the “Confidential to Editor” section, and submit your "Accept" recommendation.

Reviewer #1: (No Response)

Reviewer #3: (No Response)

**Response 1.** Thank you for the constructive feedback.

2. Is the manuscript technically sound, and do the data support the conclusions?

The manuscript must describe a technically sound piece of scientific research with data that supports the conclusions. Experiments must have been conducted rigorously, with appropriate controls, replication, and sample sizes. The conclusions must be drawn appropriately based on the data presented.

Reviewer #1: Yes

Reviewer #3: Yes

Response 2. Thank you.

 3. Has the statistical analysis been performed appropriately and rigorously?

Reviewer #1: Yes

Reviewer #3: Yes

Response 3. Thank you.

4. Have the authors made all data underlying the findings in their manuscript fully available?

The PLOS Data policy requires authors to make all data underlying the findings described in their manuscript fully available without restriction, with rare exception (please refer to the Data Availability Statement in the manuscript PDF file). The data should be provided as part of the manuscript or its supporting information, or deposited to a public repository. For example, in addition to summary statistics, the data points behind means, medians and variance measures should be available. If there are restrictions on publicly sharing data—e.g. participant privacy or use of data from a third party—those must be specified.

Reviewer #1: Yes

Reviewer #3: No

Response 4. As required, both data sets (genotypic and anthracnose severity data) are provided as supplementary Tables 1 and 2.

5. Is the manuscript presented in an intelligible fashion and written in standard English?

PLOS ONE does not copyedit accepted manuscripts, so the language in submitted articles must be clear, correct, and unambiguous. Any typographical or grammatical errors should be corrected at revision, so please note any specific errors here.

Reviewer #1: Yes

Reviewer #3: Yes

Response 5. Thank you.

6. Review Comments to the Author

Please use the space provided to explain your answers to the questions above. You may also include additional comments for the author, including concerns about dual publication, research ethics, or publication ethics. (Please upload your review as an attachment if it exceeds 20,000 characters)

Reviewer #1: Dear author,

Reviewer #1:

1- The revised version still having error with the reference and gramatical. Some edits that were red in revised version are not integrated in the clean version. Please verify the whole document.

Eg. In addition, [9] examined…………but in the edit section look that should be fixed by In addition, Cruet-Burgos et al. [9].

**Response 1**. Thank you for the feedback. As advised, the document is reviewed for grammatical and reference errors.

This reference is corrected in text: In addition, [9] examined…………is replaced with In addition, Cruet-Burgos et al. [9].

2- FarmCPU don’t use kinship. Please delete the sentence in Mat and Met.

**Response 2**. Accepted. Deleted from the Materials and Methods

3- The 20 Kb window is arbitrary and large to identified candidate genes. If you don’t have information about how large is the LD block region (I was expecting that as an answer), you need to be carefully in how to select candidate genes. I suggest you should mention: 1) the physical distance among the associate SNP and candidate gene, 2) Are any other SNP close to these candidate genes?

**Response 3**. Thank you for the insight.

At a physical distance of 51.4 kbp, the fitted LOESS curve intersected with the critical LD value. The LD values below this threshold were assumed to be due to physical linkage among inter-SNP pairs. However, in the present association panel, the 20 kb (above and below the significant SNPs) window, was chosen to be within the anticipated window of LD decay. The blue line is the trend line of nonlinear regressions against physical distance. The crucial value of r^2^ (0.1) and the LD decay value are represented by the horizontal red and vertical green lines, respectively.

4- The figure 4 is meaningless. I understand your objective but the mixed among Population structure and collection are make the figure impossible to understand. I suggest take out collection area present the disrupting of population structure from K2 to K8.

You might consider construct other type of graph to determine the relationship among population structure and collection site. Based on what you present is not association, and that is also observed in the PC graph.

**Response 4**. The population structure from K2 to K8 is removed, and a new figure has replaced this.

5- The blue line in the Fig 6 represent the significant threshold for p <0.001…no the threshold for -Log10 (P) value.You can mention -Log10 p (0.001). Same in Mat and Met’

**Response 5**. Thank you for the comment. A sentence is added in Material and Method section. An exploratory significance cutoff p <0.001 was also used and p <0.001 was also added in figure 6.

6- In the discussion, you mention about previous population structure analysis. You include the NPGS Sudan collection study [11] but not mention the population structure of NPGS Ethiopian collection (Cuevas et al. 2017. BMC Genomic) which is more related to your work.

**Response 6**. Accepted. The population structure of NPGS Ethiopian collection is mentioned in the discussion.

7- I think the discussion still need to be improved. At least I suggest you could explain: 1) why the population structure you found (8 populations) do not have association with the collection site of the samples.

**Response 7**. The population structure was independent of the collection sites, given the higher chance of genetic admixtures due to open-pollination that vary from 5 to 15% in sorghum genotypes (ADD REFERENCE).

Reviewer #3: The Manuscript entitled "Genome-wide association analysis of anthracnose resistance in sorghum [Sorghum bicolor (L.) Moench]" well written except discussion part. In the discussion, Please provide the information on how the identified 4 markers can be utilised for the resistance breeding program, which is key to the readers. Moreover, the author need to do correction, whereever marked. Furthermore, please provide the information on disease scoing part in details and attach the scoring details for each entry as supplementary table. However, the minor revision is needed for the publication acceptance.

**Response**. Thank you for the encouraging comment. This is concisely summarized in the discussion indicating the value and use of the 4 new markers as follows:

“The anthracnose resistance candidate genes identified in the present study will be validated across multiple seasons and locations to serve as ideal molecular markers for anthracnose resistance breeding programs. The novel genes could be stacked into anthracnose susceptible sorghum lines through marker-assisted or recurrent selection method. A combination of novel QTLs would render durable resistance to sorghum anthracnose.”

 6. PLOS authors have the option to publish the peer review history of their article ([what does this mean?](https://journals.plos.org/plosone/s/editorial-and-peer-review-process#loc-peer-review-history)). If published, this will include your full peer review and any attached files.

**Response 6**. Yes

If you choose “no”, your identity will remain anonymous but your review may still be made public.


**Do you want your identity to be public for this peer review?** For information about this choice, including consent withdrawal, please see our [Privacy Policy](https://www.plos.org/privacy-policy).

Reviewer #1: No

Reviewer #2: No

[NOTE: If reviewer comments were submitted as an attachment file, they will be attached to this email and accessible via the submission site. Please log into your account, locate the manuscript record, and check for the action link "View Attachments". If this link does not appear, there are no attachment files.]

While revising your submission, please upload your figure files to the Preflight Analysis and Conversion Engine (PACE) digital diagnostic tool, <https://pacev2.apexcovantage.com/>. PACE helps ensure that figures meet PLOS requirements. To use PACE, you must first register as a user. Registration is free. Then, login and navigate to the UPLOAD tab, where you will find detailed instructions on how to use the tool. If you encounter any issues or have any questions when using PACE, please email PLOS at [figures@plos.org](mailto:figures@plos.org). Please note that Supporting Information files do not need this step.
